# Digital Cognitive Behavioral Therapy for Insomnia Using a Smartphone Application in China: A Pilot Randomized Clinical Trial

**DOI:** 10.1001/jamanetworkopen.2023.4866

**Published:** 2023-03-27

**Authors:** Cheng Zhang, Yuxuan Liu, Xiaoming Guo, Yanan Liu, Yane Shen, Jing Ma

**Affiliations:** 1 Department of Respiratory and Critical Care Medicine, Peking University First Hospital, Beijing, China; 2 Department of General Medicine, Pingan Hospital, Beijing, China; 3 Department of Neurology, Peking University First Hospital, Beijing, China

## Abstract

**Importance:**

Digital cognitive behavioral therapy for insomnia (DCBT-I) requires adaptation to different sociocultural contexts. Moreover, studies comparing DCBT-I and sleep education in the same operating interface are lacking.

**Objective:**

To investigate the efficacy of a smartphone-based Chinese culture–adapted DCBT-I application (app) for insomnia compared with sleep education using the same app.

**Design, Setting, and Participants:**

This was a single-blinded, randomized clinical trial conducted from March 2021 to January 2022. Screening and randomization were conducted at Peking University First Hospital. Follow-up visits were performed online or in the same hospital. After assessing for eligibility, eligible participants were enrolled and allocated (1:1) to DCBT-I or sleep education groups. Data were analyzed from January to February 2022.

**Interventions:**

A Chinese smartphone-based app with the same interface was used in both DCBT-I and sleep education groups over 6 weeks, with 1-, 3-, and 6-month follow-ups.

**Main Outcomes and Measures:**

The primary outcome was Insomnia Severity Index (ISI) scores with the intention-to-treat principle. Secondary and exploratory outcomes included sleep diary measures; self-reported scales assessing dysfunctional beliefs about sleep, mental health, and quality of life; and smart bracelet measures.

**Results:**

Of 82 participants (mean [SD] age, 49.67 [14.49] years; 61 [74.4%] females), with 41 randomized to sleep education and 41 randomized to DCBT-I; 77 participants completed the 6-week intervention (39 participants in the sleep education group and 38 participants in the DCBT-I group; full analysis data set) and 73 completed the 6-month follow-up (per protocol data set). Mean (SD) ISI scores in the DCBT-I group were significantly lower than those in the sleep education group after the 6-week intervention (12.7 [4.8] points vs 14.9 [5.0] points; Cohen *d* = 0.458; *P* = .048) and at the 3-month follow-up (12.1 [5.4] points vs 14.8 [5.5] points; Cohen *d* = 0.489; *P* = .04). There were significant improvements from before to after the intervention for both the sleep education and DCBT-I groups, with large effect sizes(sleep education: *d* = 1.13; DCBT-I: *d* = 1.71). Some of the sleep diary measures and self-reported scales showed more improvements in the DCBT-I group than sleep education group, such as total sleep time (mean [SD]: 3 months, 403.9 [57.6] minutes vs 363.2 [72.3] minutes; 6 months, 420.3 [58.0] minutes vs 389.7 [59.4] minutes) and sleep efficiency (mean [SD]: 3 months, 87.4% [8.3%] vs 76.7% [12.1%]; 6 months, 87.5% [8.2%] vs 78.1% [10.9%]).

**Conclusions and Relevance:**

In this randomized clinical trial, the smartphone-based Chinese culture-adapted DCBT-I improved insomnia severity compared with sleep education. Future multicenter clinical trials with large sample sizes are needed to validate its effectiveness in the Chinese population.

**Trial Registration:**

ClinicalTrials.gov Identifier: NCT04779372

## Key Points

**Question:**

Can a digital cognitive behavioral therapy for insomnia (DCBT-I) application reduce insomnia severity in the Chinese cultural context?

**Findings:**

In this was single-blind randomized clinical trial including 82 participants, use of a smartphone-based and Chinese culture-adapted DCBT-I app significantly decreased Insomnia Severity Index scores compared with sleep education using the same app.

**Meaning:**

These findings suggest that the Chinese version of DCBT-I had good efficacy in reducing insomnia severity in the Chinese cultural context.

## Teaser

This randomized clinical trial investigates the efficacy of a smartphone-based Chinese culture–adapted digital cognitive behavioral therapy for insomnia application compared with sleep education in reducing insomnia severity.

## Introduction

Insomnia is a common sleep problem^1^ that affects health worldwide.^2,3,4^ The combined global prevalence of insomnia is approximately 15%^5^ and more than 200 million people in China experience insomnia. Cognitive behavioral therapy for insomnia (CBT-I) is the current recommended first-line treatment for insomnia among adults.^6,7,8,9^ However, traditional face-to-face CBT-I is inadequate in China owing to a large number of individuals with insomnia, the time-consuming process, and the relative shortage of professional therapists. Fully self-service digital CBT-I (DCBT-I) applications (apps) have been developed over the past decade,^10,11^ and they have been established to be effective in patients with insomnia in Western countries.^11,12,13,14,15,16^ Studies have shown that DCBT-I can significantly reduce the severity of insomnia compared with a waiting list control group^17^ or sleep education group.^15,18,19^ DCBT-I provides an opportunity to promote CBT-I in China, as it can offer homogeneous treatment services conveniently without limitation of personnel and location. However, insomnia and the educational nature of CBT-I are closely related to sociodemographic and cultural factors.^20,21,22^ It is unclear whether such an approach would be effective in Chinese culture.

Psychological treatments must be appropriate to the cultures in which they are used,^23^ and previous studies have shown that culturally adapted psychotherapy is superior to conventional psychotherapy.^24^ Thus, in this study, a DCBT-I tailored to the Chinese cultural context was used, including the application of local language expressions, introducing some localized activities, such as traditional Chinese calligraphy, and providing the content that is consistent with Chinese lifestyle and patterns of cognition. Given China’s large population and uneven regional development but high smartphone penetration, we developed a smartphone-based CBT-I app for easy access. The communication interface used a convenient and friendly question-and-answer format, simulating the process of consultation. Users could access the application at any convenient time and interact with it for approximately 10 to 15 minutes every day.

On the other hand, studies of DCBT-I and controls using the same interface were lacking. Patient education content in previous studies was usually obtained from a website, and users could access this content at any time during the intervention.^11,15,19,25,26,27^ It is unclear whether DCBT-I would have a more pronounced effect than sleep education given the same frequency and format of intervention.

Insomnia Severity Index (ISI) scores and sleep diaries have been recommended for assessing the severity of insomnia and subjective sleep quality for insomnia studies.^28^ Self-reported scales were also applied in this study to assess participants’ beliefs and attitudes regarding sleep, anxiety, depression, and quality of life. In addition, to record objective sleep measures, wearable devices (smart bracelets) were also applied. Therefore, the purpose of this study was to investigate whether a smartphone-based customized Chinese DCBT-I is effective for individuals in China with insomnia. The control group was the sleep education group using the same app.

## Methods

This pilot randomized clinical trial (RCT) was approved by the Peking University First Hospital human ethics committee. Written informed consent was obtained from each participant. The trial protocol and statistical analysis plan are provided in Supplement 1. This study followed the Consolidated Standards of Reporting Trials (CONSORT) reporting guideline.

## Participants

All participants had to fulfill the following inclusion criteria: (1) age at least 18 years; (2) meet the diagnostic criteria for chronic insomnia disorder according to the *International Classification of Sleep Disorders, Third Edition* (*ICSD-3*), including problems with falling asleep or maintaining sleep for at least 3 months (≥3 times per week) with impaired daytime functioning, despite the availability of sleep opportunities; (3) ISI score greater than 14 points; (4) other sleep or psychiatric disorders, if present, must be stable and require no medication; (5) proficient in the use of a smartphone app and comfortable reading, filling out the electronic questionnaire, communicating, and fully understanding the content; and (6) sign the informed consent form. Participants were excluded if any of the following exclusion criteria were met: (1) presence of shift work, alcohol abuse, substance abuse, significant suicidal ideation, bipolar disorder, aggressive behavior, mania, or schizophrenia; (2) presence of a diagnosed significant physical illness that interferes with sleep, such as cranial disease or trauma, cancer pain, unstable angina, or uncontrolled cardiac insufficiency; (3) current or previous treatment with CBT-I; (4) Epworth Sleepiness Scale score 12 points or higher; and (5) did not provide informed consent.

### Setting and Design

Participants were enrolled via recruitment advertisements and sleep clinic recruitment in Peking University First Hospital from March 2021 to January 2022. Participants were assigned random numbers after completing at least a 3-day baseline sleep diary for either DCBT-I (intervention group) or sleep education only (control group). Visits were conducted on completion of the 6-week program and at 1, 3, and 6 months thereafter. Prior to each visit, participants were asked to complete a 1-week sleep diary and use a smart bracelet (Xiaomi Bracelet 3 NFC) for data collection . On the day of the visit, participants completed questionnaires on the ISI, Dysfunctional Beliefs and Attitudes About Sleep 16-item scale (DBAS-16), Fatigue Severity Scale, Generalized Anxiety Disorder Assessment 7-item (GAD-7), and Patient Health Questionnaire 9-item (PHQ-9), and Short-Form 12-item Health Survey (SF-12).

Screening and randomization were conducted in the Peking University First Hospital. Follow-up visits were performed online or in the same hospital.

### Intervention

DCBT-I (intervention group) participants received a 6-week self-guided DCBT-I provided by a smartphone-based app called *resleep* that included sleep hygiene education, stimulation control, relaxation therapy, sleep restriction, and cognitive therapy. Sleep education group (control group) participants received sleep hygiene education and information on stimulus control, with relevant content overlapping with the corresponding modules within DCBT-I. The study had no requirement regarding patients’ medication status. Details on the content of the DCBT-I app are provided in eTable 1 and eFigure 1 in Supplement 2.

### Randomization and Masking

Randomization was computer-generated, and the random number tables were maintained by a staff member of Peking University First Hospital who was not involved in this study. Participants were assigned to DCBT-I and sleep education groups in a 1:1 ratio according to random numbers. Participants were masked for group allocation.

### Outcomes and Measures

#### Primary Outcome

The primary outcome was ISI total scores at treatment completion and follow-ups. The ISI is a questionnaire commonly used to evaluate the severity of insomnia.^29^ Severity is classified into 4 categories according to the total score: no clinically significant insomnia (0-7 points), subthreshold insomnia (8-14 points), moderate clinical insomnia (15-21 points), and severe clinical insomnia (22-28 points).

#### Secondary Outcomes

Secondary outcomes included online sleep diary measures of sleep latency, sleep efficiency, total sleep time, and number and duration of awakenings during sleep. Participants were asked to complete sleep diaries daily during the 6-week intervention and 1 week before each follow-up visit.

#### Exploratory Outcomes

We also assessed a number of exploratory outcomes, including sleep-related attitudes and beliefs, tiredness and its affects on daily activities, anxiety, depression, and quality of life. Sleep-related attitudes and beliefs were assessed with DBAS-16.^16,30^ The questionnaire contains 16 items reflecting different beliefs and attitudes concerning sleep. Each question is scored from 0 to 10, with a total score range of 0 to 160. A higher score reflects greater dysfunctional beliefs about sleep.

Tiredness and how it affects daily activities were assessed using the Fatigue Severity Scale,^31^ containing 9 items. Each response is given a score between 1 and 7, with 7 representing a strong agreement. The range of possible scores is 9 to 63, with higher scores indicating more tiredness. The scale has showed good consistency and reproducibility.^32^

Anxiety was measured using the GAD-7, a simple and reliable anxiety screening tool.^33^ It contains 7 questions, each of which receives a score of 0 to 3. The overall score is between 0 and 21, with higher scores indicating more anxiety.

Depression was assessed using the PHQ-9,^34^ a short, reliable, and valid self-assessment tool for depressive disorders that may be used to diagnose depression and gauge the severity of its symptoms. It contains 9 items, with total score range of 0 to 27 and higher scores indicating worse depression.

For quality of life assessment, a streamlined quality of life survey called the SF-12 was developed using the SF-36 scale.^35^ The SF-12 uses 12 items to evaluate 8 aspects of a person’s subjective physical and mental health. Raw scores are converted using a standardized scoring method. The SF-12 is divided into physical component summary and mental component summary scores. Higher scores indicate higher levels of quality of life. Smart bracelets collected data on total sleep time and time of waking up during sleep.

### Statistical Analysis

Using ISI as the main outcome and intervention and control group allocated as 1:1, the difference in ISI improvement after treatment (DCBT-I vs controls) was estimated to be approximately 6 with an SD of approximately 8. Based on previous literature,^11,36^ we set at α = .05 (2-sided test), with a certainty of 1 − β = 80%. The sample size of 29 participants per group was calculated using PASS sample size software version 11.0 (NCSS Statistical Software). Based on the assumption of a 30% shedding rate reported in previous studies, a total sample size of 2 × (29 / 0.7) ≈ 82 participants was required, with 41 participants included in each group.

SPSS Statistics for Windows, version 23 (IBM) was used for statistical analysis. The main outcome indicators were analyzed using the intention-to-treat (ITT) principle. The full analysis data set was defined as patients who completed at least 6 weeks of intervention. Missing ISI values were replaced by Linear Interpolation. Continuous data were expressed as mean and SD, and an independent samples *t* test was used to compare the 2 groups. Categorical data were described statistically using frequency (percentage), and the χ^2^ test or Fisher exact test was used for comparison between groups. Differences were considered statistically significant at 2-sided *P* < .05. Effect sizes were expressed as Cohen *d*, with a large effect size defined as *d* greater than 0.8; medium effect size, 0.5 to 0.8; and small effect size, 0.2 to 0.5. Data were analyzed from January to February 2022.

## Results

**Figure 1.**
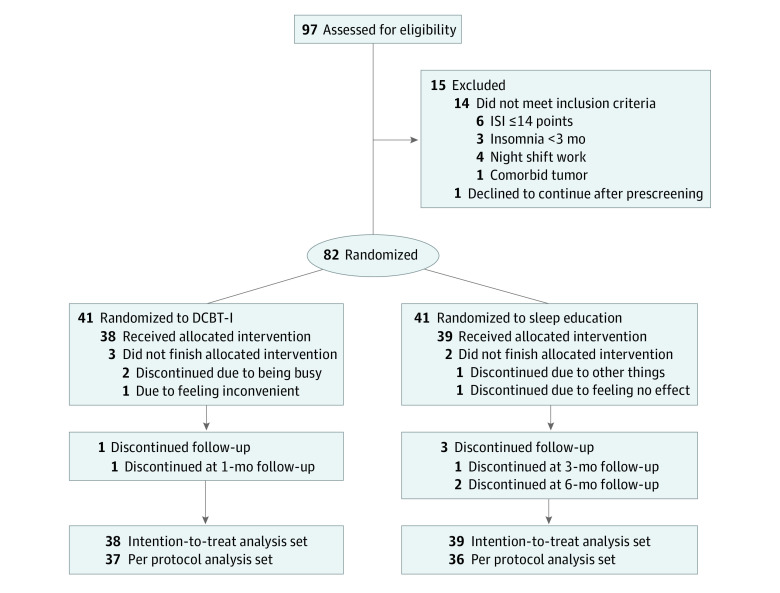
Participant Recruitment Flowchart DCBT-I indicates digital cognitive behavioral therapy for insomnia; ISI, Insomnia Severity Index.

In total, 97 individuals were screened for eligibility, of whom 6 had ISI of 14 or lower, 3 had insomnia for less than 3 months, 4 were engaged in night shift work, 1 reported a comorbid tumor, and 1 failed to attend screening after enrollment. A total of 82 participants (mean [SD] age, 49.67 [14.49] years; 61 [74.4%] females) were enrolled and were equally assigned to the sleep education and DCBT-I groups; 5 individuals did not complete the 6-week treatment, 1 individual did not complete the 1-month follow-up, 1 individual did not complete the 3-month follow-up, and 2 individuals did not complete the 6-month follow-up. Thus, 77 individuals (mean [SD] age, 50.13 [13.49] years; 56 females) completed the 6-week program and 73 patients completed the entire clinical trial. Therefore, 77 participants, including 39 participants in the sleep education group and 38 participants in the DCBT-I group, were included in the full analysis data set. At baseline, there was no difference in ISI scores between groups (mean [SD] ISI score: sleep education, 20.9 [3.3]; DCBT-I: 20.8 [3.7]). The participant recruitment flowchart is shown in Figure 1. Baseline demographic information is shown in Table 1.

**Table 1.** Baseline Participant Characteristics

Characteristic	Participants, No. (%)	
	Sleep Education (n = 39)	DCBT-I (n = 38)
Age, mean (SD), y	50.6 (14.1)	49.6 (13.0)
Height, mean (SD), cm	165.5 (9.3)	165.1 (6.7)
Weight, mean (SD), kg	65.0 (12.1)	64.4 (12.1)
BMI, mean (SD)	23.6 (3.2)	23.5 (3.5)
Sex		
Male	13 (33.3)	8 (21.1)
Female	26 (66.7)	30 (78.9)
Education level		
≤Primary school	2 (5.1)	0
Secondary school	9 (23.1)	7 (18.4)
University	21 (53.8)	24 (63.2)
≥Graduate school	7 (17.9)	7 (18.4)
Marital status		
Married	31 (79.5)	32 (84.2)
Single	8 (20.5)	6 (15.8)
Employment status		
Full-time	14 (35.9)	18 (47.4)
Part-time	3 (7.7)	2 (5.3)
Retired	18 (46.2)	16 (42.1)
Full-time homemaker	2 (5.1)	1 (2.6)
Unemployed	1 (2.6)	0
Full-time student	1 (2.6)	0
Missing	0	1 (2.6)
Living status		
Living alone	5 (12.8)	5 (13.2)
Living with parents	3 (7.7)	2 (5.3)
Living with adult children	1 (2.6)	2 (5.3)
Living with partner	11 (28.2)	12 (31.6)
Living with partner and children	16 (41.0)	16 (42.1)
Other	3 (7.7)	1 (2.6)
Coffee drinking		
Never	26 (66.7)	23 (60.5)
<1 time/d	5 (12.8)	8 (21.1)
1 time/d	6 (15.4)	3 (7.9)
≥2 times/d	2 (5.1)	4 (10.5)
Alcohol consumption		
Never	30 (76.9)	26 (68.4)
<1 time/d	7 (17.9)	9 (23.7)
1 time/d	1 (2.6)	1 (2.6)
≥2 times/d	1 (2.6)	1 (2.6)
Missing	0	1 (2.6)
Smoking status		
Never	32 (82.1)	36 (94.7)
Quit	1 (2.6)	0
<10 cigarettes/d	2 (5.1)	0
≥10 cigarettes/d	3 (7.7)	1 (2.6)
Missing	1 (2.6)	1 (2.6)
Physical exercises		
Never	8 (20.5)	13 (34.2)
<1 time/wk	4 (10.3)	2 (5.3)
1-2 times/wk	6 (15.4)	5 (13.2)
≥3 times/wk	21 (53.8)	18 (47.4)

Abbreviations: BMI, body mass index (calculated as weight in kilograms divided by height in meters squared); DCBT-I, digital cognitive behavioral therapy for insomnia.

### ISI Scores

Comparison of ISI scores between the DCBT-I and sleep education groups found that ISI scores in the DCBT-I group were significantly lower than in the sleep education group on completion of the 6-week intervention (mean [SD] score, 12.7 [4.8] points vs 14.9 [5.0] points; *d* = 0.458; *P* = .048) and at the 3-month follow-up (mean [SD] score, 12.1 [5.4] points vs 14.8 [5.5] points; *d* = 0.489; *P* = .04) (Table 2). At the 6-month follow-up, there was no significant difference between the DCBT-I group and sleep education group in ISI scores (mean [SD] score, 11.5 [5.3] points vs 14.0 [5.5] points; *d* = 0.454; *P* = .05). The results of the comparison of ISI scores between groups using the per-protocol (PP) set are shown in eTable 2 in Supplement 2. A regression model with a random slope was used to quantify the group × time interactions. There were significant group × time interaction effects between groups at intervention completion (difference, −2.16; 95% CI, −4.21 to −0.11), 3 months (difference, −2.77; 95% CI, −4.84 to −0.70), and 6 months (difference, −2.61; 95% CI, −4.69 to −0.52) (eFigure 2 in Supplement 2).

**Table 2.** The Comparisons of ISI Scores of DCBT-I vs Sleep Education

Period	ISI score, mean (SD)		*t*	*P* value	Cohen *d* (95% CI)
	Sleep education	DCBT-I			
Baseline	20.9 (3.3)	20.8 (3.7)	0.102	.92	NA
Postintervention	14.9 (5.0)	12.7 (4.8)	2.011	.048	0.458 (0.004 to 0.910)
1-mo follow-up	14.5 (5.0)	12.9 (5.1)	1.402	.17	0.318 (−0.133 to 0.767)
3-mo follow-up	14.8 (5.5)	12.1 (5.4)	2.147	.04	0.489 (0.034 to 0.941)
6-mo follow-up	14.0 (5.5)	11.5 (5.3)	1.979	.05	0.454 (0.000 to 0.905)

Abbreviations: DCBT-I, digital cognitive behavioral therapy for insomnia; ISI, Insomnia Severity Index; NA, not applicable.

**Figure 2.**
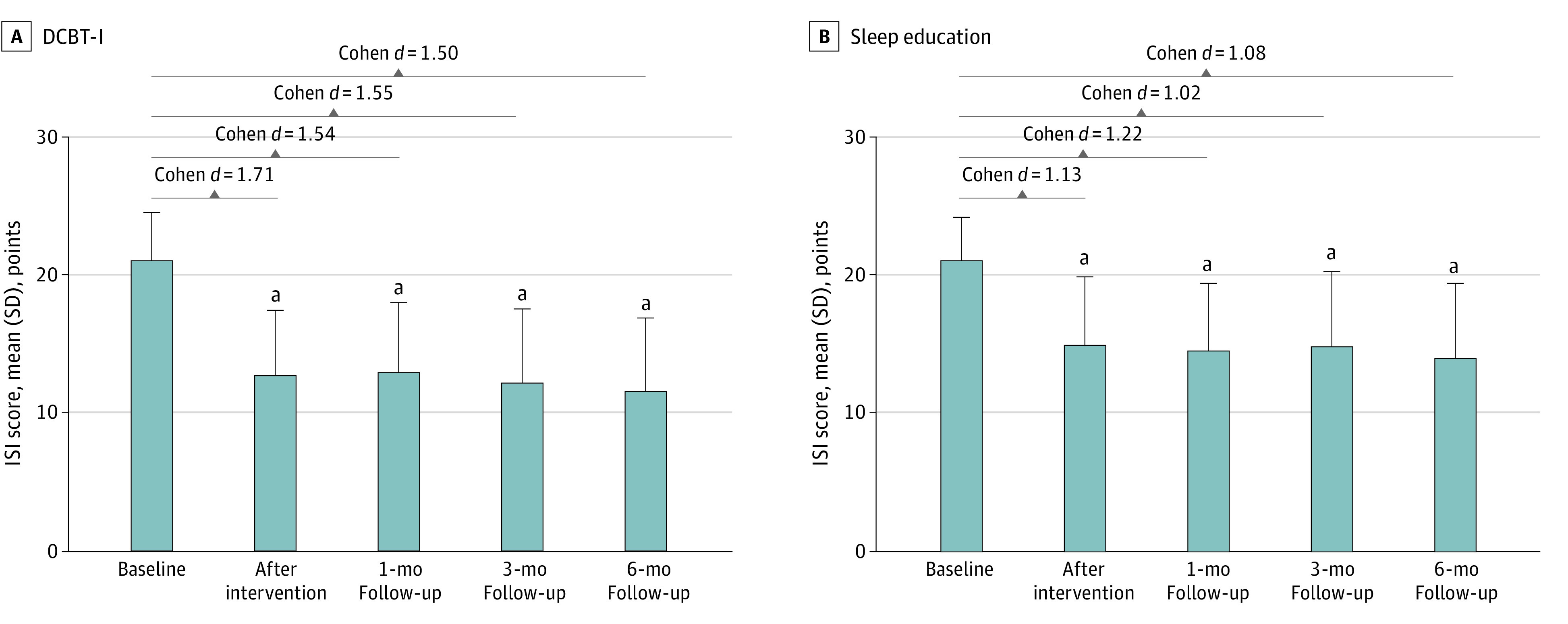
Insomnia Severity Index (ISI) Scores at Each Time Point Compared With the Baseline DCBT-I indicates digital cognitive behavioral therapy for insomnia. ^a^Statistically significantly different from baseline.

There were significant improvements after treatment for both sleep education and DCBT-I groups at intervention completion and follow-ups, with a large effect size (sleep education: *d* = 1.02 to 1.22; DCBT-I: *d* = 1.50 to 1.71) (Figure 2). Participants with ISI reduction of at least 8 points were defined as ISI responders.^16^ In the DCBT-I group, 25 participants (65.8%) were classified as responders at intervention completion, and 24 participants (63.2%) were classified as responders at 3 months, which was significantly higher than among the sleep education group: 12 participants (30.8%) were responders after intervention completion and 14 participants (35.9%) were responders at 3 months. An ISI score of less than 8 points was used to define ISI remission.^29^ There were no statistically significant differences for remission between groups (eTable 3 in Supplement 2).

### Sleep Diary Measures

The sleep efficiency, sleep latency, number of awakenings during sleep, and wake time after sleep onset of the DCBT-I group were significantly improved compared with the sleep education group at intervention completion. The DCBT-I group had significantly higher total sleep time than the sleep education group at the 3-month (mean [SD], 403.9 [57.6] minutes vs 363.2 [72.3] minutes) and 6-month (mean [SD], 420.3 [58.0] minutes vs 389.7 [59.4] minutes) follow-ups. Similarly, sleep efficiency was significantly better in the DCBT-I group than the sleep education group at the 3-month (mean [SD], 87.4% [8.3%] vs 76.7% [12.1%]) and 6-month (mean [SD], 87.5% [8.2%] vs 78.1% [10.9%]) follow-ups (Table 3).

**Table 3.** Comparisons of the Sleep Diary Measures Between the DCBT-I and Sleep Education and Their Effect Sizes

Sleep diary measure	Mean (SD)		*t*	*P* value	Cohen *d* (95% CI)
	Sleep education	DCBT-I			
**Sleep onset latency, min**					
Baseline	42.8 (30.5)	61.6 (55.8)	−1.846	.07	NA
Postintervention	34.2 (25.2)	19.5 (23.7)	2.636	.01	0.601 (0.142 to 1.056)
1-mo follow-up	33.5 (28.9)	31.1 (35.2)	0.328	.74	0.076 (−0.374 to 0.525)
3-mo follow-up	40.7 (39.7)	26.4 (19.6)	1.973	.05	0.456 (−0.004 to 0.913)
6-mo follow-up	34.2 (26.7)	26.3 (21.5)	1.39	.17	0.325 (−0.138 to 0.786)
**Sleep efficiency, %**					
Baseline	68.9 (15.6)	68.9 (16.0)	−0.02	.98	NA
Postintervention	76.9 (14.9)	87.9 (9.5)	−3.853	<.001	−0.878 (−1.344 to −0.407)
1-mo follow-up	78.8 (11.4)	86.1 (9.7)	−3.043	.003	−0.698 (−1.160 to −0.232)
3-mo follow-up	76.7 (12.1)	87.4 (8.3)	−4.467	<.001	−1.032 (−1.511 to −0.546)
6-mo follow-up	78.1 (10.9)	87.5 (8.2)	−4.149	<.001	−0.971 (−1.454 to −0.483)
**Number of awakenings during sleep**					
Baseline	2.0 (1.3)	1.9 (1.2)	0.16	.87	NA
Postintervention	1.5 (1.2)	1.0 (0.8)	2.304	.02	0.525 (0.069 to 0.978)
1-mo follow-up	1.4 (1.1)	1.2 (0.8)	0.892	.38	0.205 (−0.247 to 0.655)
3-mo follow-up	1.5 (1.3)	1.3 (0.9)	0.564	.57	0.130 (−0.323 to 0.583)
6-mo follow-up	1.6 (1.3)	1.4 (1.1)	0.639	.53	0.150 (−0.310 to 0.609)
**Wake time after sleep onset, min**					
Baseline	41.6 (35.4)	33.8 (26.6)	1.099	.28	NA
Postintervention	28.5 (25.9)	15.1 (15.4)	2.749	.007	0.627 (0.167 to 1.082)
1-mo follow-up	23.8 (23.9)	19.0 (20.3)	0.924	.36	0.213 (−0.241 to 0.667)
3-mo follow-up	26.1 (27.6)	19.4 (25.4)	1.086	.28	0.251 (−0.205 to 0.704)
6-mo follow-up	28.8 (30.2)	14.6 (14.8)	2.548	.01	0.596 (0.125 to 1.064)
**Total sleep time, min**					
Baseline	317.0 (83.4)	332.1 (74.5)	−0.839	.40	NA
Postintervention	357.8 (67.3)	368.0 (71.8)	−0.642	.52	−0.146 (−0.593 to 0.302)
1-mo follow-up	366.8 (62.4)	391.9 (71.8)	−1.627	.11	−0.373 (−0.826 to 0.082)
3-mo follow-up	363.2 (72.3)	403.9 (57.6)	−2.695	.009	−0.622 (−1.084 to −0.157)
6-mo follow-up	389.7 (59.4)	420.3 (58.0)	−2.231	.03	−0.522 (−0.987 to −0.054)

Abbreviation: NA, not applicable.

### Exploratory Outcomes

#### Self-reported Scale Scores

The mental component scores of the SF-12, PHQ-9, and GAD-7 showed more improvement in the DCBT-I group than in the sleep education group at the 3-month follow-up (eTable 4 in Supplement 2). High internal consistency coefficients were obtained for PHQ-9 (Cronbach α = .841) and GAD-7 (Cronbach α = .904) in this study by analyzing the baseline data.

#### Subjective Sleep Quality Parameters Recorded by Smart Bracelet

The total sleep time recorded by the smart bracelet of the DCBT-I group was significantly higher than that of the sleep education group at the 3-month follow-up (mean [SD], 448.4 [71.3] minutes vs 395.8 [91.8] minutes) (eTable 5 in Supplement 2). Other parameters recorded by the smart bracelet were not significantly different between groups.

#### Sleep Medication

The frequency of sleep medication use at baseline was similar in the DCBT-I and sleep education groups. There were no significant differences in the frequency of sleep drug use between groups during treatment (χ^2^ = 1.979; *P* = .37) or during follow-up (χ^2^ = 0.039; *P* = .98).

## Discussion

This pilot randomized clinical trial found that the smartphone-based, Chinese culture-adapted DCBT-I app reduced the severity of insomnia (measured with ISI scores) and improved sleep quality (assessed via sleep diary) compared with a sleep education control provided via the same app. The DCBT-I app has several features to adapt to Chinese culture, communication, and lifestyle. Regarding language expressions, we applied a contextually appropriate and easily understandable verbal communication style to facilitate interaction with participants and foster a better understanding of the treatment. Furthermore, the behavioral and cognitive guidance was provided in a manner congruent with the Chinese lifestyle and introduced some localized activities. For example, in the stimulus control section, some activities, such as knitting sweaters and traditional Chinese calligraphy, were added to the suggested activities to be performed by the participants outside of their beds when they could not sleep. In the relaxation section, the study app arranged the progressive muscle relaxation practices at noon instead of in the afternoon, when most people in China are accustomed to napping, since taking a nap might reduce sleep motivation at night. In the cognitive and stimulus control section, a traditional Chinese concept holds that “Zi Wu sleep” is beneficial to health. Zi Wu sleep suggests that people need to go to sleep for the night by 11 pm, with deep sleep occurring at least until 1 am (Zi sleep), and have a nap between 11 am and 1 pm (Wu sleep). Sleep behaviors for many people in China are influenced by this concept. They may go to bed at 11 pm without feeling sleepy. Therefore, the study app emphasized the principles and effects of sleeping restriction to correct misconceptions and facilitate the CBT-I process.

The chatbot format is another prominent feature of the app. The communication interface of the chatbot used a convenient and friendly question-and-answer format, rather than using full pages of text or completing forms. The study app presents as a virtual sleep therapist by simulating the language expressions of a real-world therapist through a chat tone, which could facilitate patients’ trust and acceptance of the intervention. The information collected for the sleep diary and all interventions are given in this kind of chatbot form.

This study established a sleep education control that used the same app as the intervention group. To our knowledge, this is the first RCT in which the control group received sleep education provided using the same interface as an intervention. The use of the same app allowed this study to be single-blinded. In previous studies, content for the sleep education control was obtained from a website, which was available all at once, and a user could access the full site content at any time during the intervention.^11,15,19,25,26,27^ In contrast, for this study, our control group was provided 1 specific aspect of information daily through the app. In addition to information on sleep hygiene, information on stimulus control was also provided. The sleep education group was also asked to keep a daily sleep diary during the 6-week program, as was the DCBT-I group. Any or all these factors may have been the cause of the significant decrease in ISI in the sleep education group from baseline to intervention completion, with a large effect size, indicating this kind of sleep education may also alleviate the severity of insomnia. However, the DCBT-I group significantly outperformed the control group in improved insomnia severity and subjective sleep quality, demonstrating the efficacy of DCBT-I.

Sleep diaries are the main method for subjective sleep quality assessment. Similar to previous studies,^11,15^ this study found that the DCBT-I group had better outcomes than the control group in terms of sleep efficiency, sleep latency, number and duration of nighttime awakenings, and total sleep time, as recorded by the sleep diary. An exploratory analysis suggested that DCBT-I might be also superior to the sleep education group in terms of reducing depression and anxiety, improving quality of life (as detected by self-reported scales), and some of objective sleep quality measures (as detected by smart bracelet). However, these are preliminary explorations, and further studies with large samples are needed to confirm these findings.

This study had good adherence and a low rate of attrition, with 93.9% of participants completing treatment and 89.0% of participants completing the 6-month follow-up. Previous studies have reported attrition rates of 20 to 45%. For example, a 2016 study^34^ reported that only 51% of the participants completed the DCBT-I and 44% of participants completed the 6-month follow-up. Our higher adherence and lower attrition may be related to the interface of the app, which used a chatbot format, and the content and logic of the app design encouraging participants to continue. On the other hand, the fact that we performed face-to-face screening and enrollment may have also contributed to increased adherence.

### Limitations

This study has several limitations. First, this is a single-center, preliminary RCT study with women being overrepresented (approximately 70% of the sample). A larger sample size is needed to validate our findings. Second, the objective evaluation of sleep was performed using a consumer-grade smart bracelet rather than with medical actigraphy, and the quality of sleep parameter measures varies between these products. Third, participants with other sleep disorders, such as periodic limb movement disorder and obstructive sleep apnea, were not excluded from this study. Additionally, there may be a population selection bias, as participants were mainly from Beijing, China.

## Conclusions

In this pilot randomized clinical trial, the culturally adapted Chinese smartphone-based DCBT-I app reduced the severity of insomnia and improved sleep quality compared with a sleep education control. These findings suggest that culturally adapted DCBT-I is suitable for the large population of patients with insomnia in China. Future multicenter clinical trials with large sample sizes are needed to validate its effectiveness in the Chinese population.

## Notes

Supplement 1.
Trial Protocol and Statistical Analysis Plan

Supplement 2. **eTable 1.** Contents of Each Phase of DCBT-I App **eTable 2. **Comparisons of DCBT-I vs Sleep Education ISI Scores in PP Analysis **eTable 3.** Comparison of ISI Response and Remission Between the DCBT-I and Sleep Education Groups **eTable 4.** Comparisons of Self-reported Scales Between the DCBT-I and Sleep Education Groups **eTable 5.** Comparisons of Smart Bracelet Measures Between the DCBT-I and Sleep Education Groups **eFigure 1.** Differences in ISI Scores Between the DCBT-I and Sleep Education Groups Over Time, Estimated From a Regression Model With a Random Slope **eFigure 2.** Screenshots of the DCBT-I App

Supplement 3.
Data Sharing Statement
