# Supplementary material for: Digital Cognitive Behavioral Therapy for Insomnia Using a Smartphone Application in China: A Pilot Randomized Clinical Trial
Source: JAMA Netw Open. 2023 Mar 27;6(3):e234866. doi: 10.1001/jamanetworkopen.2023.4866 (PMC10043748; doi:10.1001/jamanetworkopen.2023.4866)
Supplement: Supplement 1. — Trial Protocol and Statistical Analysis Plan [file jamanetwopen-e234866-s001.pdf]

SUPPLEMENT 1. Trial Protocol

Project name: A randomized controlled clinical study of the effect of digital cognitive behavioral therapy for insomnia (dCBT-I) on the treatment of chronic insomnia

I. Research Overview / Background / Rationale

Insomnia is the most common sleep problem, affecting approximately 20-40% of adults worldwide, of which 9-15% meet the diagnostic criteria for insomnia disorder [i].45.4% of respondents in China have experienced varying degrees of insomnia in the past 1 month [ii].About 74% of insomniacs report insomnia lasting more than 1 year [iii].Insomnia can be divided into short-term insomnia and chronic insomnia. Short-term insomnia disorders are mostly associated with significant stressors and can mostly resolve on their own with the removal of the stressor or appropriate coping patterns, while some patients can progress to chronicity due to poor coping patterns. The diagnostic criteria for chronic insomnia disorder are: problems falling asleep or maintaining sleep for ≥3 months (≥3 times per week) with impaired daytime functioning, despite the opportunity to sleep[iv、v].Chronic insomnia disorder is difficult to treat and has far-reaching effects.

Insomnia can affect the quality of life, work efficiency and production safety, but also cause a huge social burden, research shows that the health and economic burden of patients with insomnia disorder is 11 times more than those who sleep well[vi].Chronic insomnia disorder (chronic insomnia disorder) is associated with other chronic diseases such as obesity, diabetes, hypertension, cardiovascular disease, sleep apnea, anxiety, depression, etc[i、vii、viii] , Increase the burden on society by causing indirect losses.

Currently, insomnia is treated with medication, cognitive-behavioral therapy, and a combination of the two. Although medication is fast-acting and can improve sleep in the short term, there is no long-term evidence of effectiveness[ix] , Long-term use can result in a variety of adverse effects such as increased drug tolerance and dependence, withdrawal rebound, daytime functional impairment, motor incoordination, and increased risk of falls in hospitalized elderly[x].Multiple moderate and high-quality randomized controlled studies, systematic reviews, etc.[xi、xii]Cognitive behavioral therapy for insomnia (CBT-I) has been shown to be an effective treatment for insomnia without the adverse effects similar to those of drugs. Moreover, the duration of efficacy of CBT-I is longer than that of drugs, and the positive effects of treatment can last up to 1 year and still have significant clinical significance[xiii].Therefore, CBT-I is currently recommended as the first-line treatment for chronic insomnia in adults in both Chinese and foreign authoritative guidelines[ii、xiv、xv], or in combination with drug therapy if necessary.

CBT-I is a multimodal cognitive-behavioral therapy that focuses on insomnia and includes sleep hygiene education, stimulus control, relaxation therapy, sleep restriction therapy and cognitive therapy, and typically requires 6-8 weeks of treatment. CBT-I is a therapy with an educational nature and has the advantage of providing patients with tools that can be applied in the future. However, the traditional CBT-I treatment process is complex, financially costly, and requires an experienced and well-trained therapist or physician to have sufficient benefit. While the main treatment sites for insomnia are in primary care, psychiatric-psychological clinics, and sleep clinics, the lack of qualified therapists or clinicians skilled in all treatment components makes CBT-I difficult to obtain. There are operational differences and even irregularities between practitioners that can affect the efficacy[xvi].This situation is accentuated by the large population base in China, the large number of insomnia patients, the shortage of medical personnel, and the uneven distribution of medical resources. Therefore, there is an urgent need for new high quality and affordable CBT-I implementation methods with wide coverage.

In recent years, with the popularization of information technology, Internet-based CBT-I has been established and developed to provide convenient, standardized and homogeneous treatment services for insomnia patients, breaking through the limitations of time and space. Networked CBT-I is mainly

divided into two forms: full/partial participation of therapists and full self-help without therapist participation. Full self-help is the most economical way, but it requires high technical requirements and poor compliance. Several RCT studies and meta-analyses have been conducted abroad[xvii、xviii]show , internet CBT-I can significantly reduce the severity of insomnia, improve sleep efficiency and total sleep time, enhance sleep confidence, and reduce the occurrence of depression compared with the waiting treatment group or the general sleep education group. internet CBT-I has shown its unique advantages and attracted high attention since the global novel coronavirus outbreak in the first half of 2020. Internet CBT-I in China is still in its infancy, and existing ongoing registration studies use a networked CBT-I system with psychoeducation and homework as the main interventions, with the goal of reducing the occurrence of depression and reducing the formation of related suicidal ideation in insomniacs.

The main problems with current networked CBT-I are: 1. Internet-based CBT-I in most clinical studies is implemented through a web-based platform, and there are relatively few digital CBT-I (dCBT-I) implemented using mobile device applications, which are simpler to implement, more flexible, and have a wider audience, and may be more suitable for the reality of our country where economic and cultural development is uneven but smartphones are very popular. 2. poor adherence to fully self-help CBT-I treatment in clinical studies, with a shedding rate of about 20%-45%, may be related to the fact that the software implements education through reading or video but has insufficient interactive features. 3. most web-based CBT-I implementations follow a set sequence of modules one by one, with insufficient individualization of patient response to treatment, which affects efficacy and time to achieve efficacy.

Therefore, digital CBT-I (dCBT-I) treatment based on smartphone application, using intelligent algorithms to increase interactive functions and improve educational experience, may be beneficial to solve the above web-based CBT-I bottlenecks and further accelerate symptom improvement, improve efficacy and treatment adherence. The implementation of dCBT-I through cell phone application is very suitable for China's vast geographical area, the serious imbalance of medical resources but the popularity of smart phones, and will be beneficial to the treatment of the widely distributed and large number of insomnia people in China, as well as to empower primary medical institutions to carry out standardized and effective insomnia treatment, in line with China's "Healthy China" strategic plan. Strategic planning. However, there are few mature dCBT-I in China, and there is a lack of data on the efficacy of dCBT-I for chronic insomnia patients in China.

To initially explore the effectiveness of smartphone-based dCBT-I therapy for chronic insomnia patients in China, we propose to conduct a randomized, open clinical trial study using a Chinese App "resleep" that meets the above requirements, with a general sleep education group as a parallel control group.

"Resleep" is developed by Shenzhen Zeen Health Technology Co., Ltd.With independent intellectual property rights, based on CBT-I theory, combined with algorithmic technology, we can develop individualized treatment plans for patients based on the sleep and behavioral data of a large number of people with insomnia.Unlike traditional CBT-I which is completed in 6-8 weeks through outpatient appointments, users can open the "resleep" App at any convenient time of the day and receive treatment plans by interacting with the chatbot, with each communication taking about 10-15 minutes. The program collects and analyzes the patient's sleep habits, insomnia and sleep cognition during each conversation, and then integrates the five parts of the CBT-I core curriculum step by step to give individualized guidance and complete the treatment in about 4-6 weeks. Moreover, patients' treatment response and changes in sleep data are tracked and analyzed during the treatment period, and the program is continuously adjusted. Preliminary data showed that 85% of patients had significant improvement in subjective sleep quality within two weeks, and no significant adverse effects were found.

II. Purpose of the study

To explore the effectiveness of digital CBT-I therapy based on smartphone application for chronic insomnia patients in China.

III. Research Methodology

3.1 Research Design Methodology

Randomized, parallel controlled study.

3.2 Study site for data collection

Study duration: February 2021 or after ethics committee approval - August 2022
Subject recruitment period: February 2021 or after ethics committee approval - August 2021
Data collection site:

- Demographic data, medical history, questionnaire evaluation: Sleep Monitoring Unit, Department of Respiratory Medicine, Peking University Hospital
- Sleep diary, sleep and symptom profile during treatment: App, smart bracelet

3.3 Research Subjects

3.3.1 Inclusion criteria for study subjects: all of the following were met before inclusion.

- Age ≥ 18 years old.
- Meets the diagnostic criteria for chronic insomnia disorder in the ICSD-3: Problems falling asleep or difficulty maintaining sleep for ≥3 months (≥3 times per week) despite having access to sleep, it is also accompanied by impaired daytime function.
- Insomnia Severity Index (ISI) > 14 points.
- If other sleep or psychiatric disorders are present, they need to be stable and have no requirement for medication.
- Proficient in using mobile App, comfortable reading, filling out electronic questionnaires, communicating and fully understanding the content.
- Sign the informed consent form.

3.3.2 Exclusion criteria for study subjects: ≥1 of the following were met, then they could not be enrolled.

- Any of the following conditions currently exist: shift work, alcohol abuse, substance abuse, overt suicidal ideation, bipolar disorder, aggressive behavior, mania, schizophrenia.
- Presence of a diagnosed significant physical illness that interferes with sleep, such as cranial disease or trauma, cancer pain, unstable angina, uncontrolled cardiac insufficiency, etc.
- Being or previously treated with CBT-I.
- Epworth Sleepiness Scale (ESS) score ≥ 12.
- Informed consent was not signed.

3.4 Interventions

- Intervention group: a 6-week dCBT-I implemented by a smartphone App, consisting of sleep hygiene education, stimulation control, relaxation therapy, sleep restriction therapy and cognitive therapy. The App uses chatbot to talk to subjects and individualize adjustments by algorithmically analyzing patient data and feedback.
- Control group: received sleep hygiene education and stimulus control knowledge available at routine clinic visits, with relevant content overlapping with the corresponding modules within the CBT-I.
- The study had no requirements for the medication the patient was receiving.

3.5 Research Indicators

3.5.1 Primary endpoint indicator: insomnia severity index (ISI)

3.5.2 Other study metrics:

- Online sleep diary (7d): total time in bed (TIB), total sleep time (TST), sleep efficiency (SE), and total time awake (TWT) were calculated from the sleep diary
- Smart bracelet recorded: total sleep time, number of times waking up during sleep, wake up time and sleep quality.
- Simplified Sleep Attitudes and Beliefs Questionnaire (DBAS-16)
- Fatigue Severity Scale (FSS)
- Quality of Life Questionnaire (SF-12)
- Generalized Anxiety Disorder Assessment (GAD-7)
- Patient Health Questionnaire (PHQ-9 Depression Screening)

3.5.3 Other information to be collected: age, sex, education, employment status, living and residence status, demographic data, current medical history, past medical history and medication, smoking and alcohol consumption, body mass index (BMI), etc., and adverse effects during treatment.

3.6 Research Plan

- Patients were recruited by subject recruitment advertisements and sleep clinics, interviewed in person, screened after signing informed consent, and demographic data, medical history, and clinical information were collected on the day of screening.
- Those who met the inclusion criteria were randomly assigned to the group after recording a sleep diary for at least 3 days.
- A bracelet was issued on the day of enrollment, instructions were given to download the app, and access to the appropriate group was logged in based on the randomization number. A 6-week period of dCBT-I or sleep education was started on the day of enrollment, and compliance and efficacy were monitored by investigators without intervention.
- Follow-up schedule(online/offline): at the end of the intervention, 1 month, 3 months, and 6 months after the end of the intervention.
- Study endpoint: 6 months after the end of the intervention.
- Clinical information related to study indicators included: symptoms, physical examination and medication information, scale assessments (ISI, DBAS-16, FSS, SF-12, GAD-7, PHQ-9), sleep diary, and bracelet recording of sleep indicators. The time points for collection were: baseline, at the end of the intervention, 1 month, 3 months and 6 months after the end of the intervention.
- Follow-up continued for 6 months after the end of the study (1 year after the end of the intervention) and subjects who volunteered to participate completed symptoms online scale assessments (ISI, DBAS-16, FSS, SF-12, GAD-7, PHQ-9) and recorded a 1-week sleep diary.

3.7 Sample size

This was a randomized controlled study with a parallel design, with the intervention group being the dCBT-I treatment group and the control group being the general sleep education group, with a sample size of 1:1 in both groups. using the Insomnia Severity Index (ISI) as the main study index, the difference in ISI improvement after treatment was estimated to be approximately 6 with a standard

deviation of approximately 8, based on previous literature, set at α=0.05 (two-sided test), with a certainty of 1-β=80%. The sample size of 29 cases per group was calculated using PASS 11.0 software. Based on the assumption of a 30% shedding rate reported in previous studies, a total sample size of n=2*(29/0.7) ≈ 82 cases is required, with 41 cases of study subjects included in each group.

3.8 Data Management

- Data collection: Data collection: Primary and secondary outcomes at baseline and follow-up were completed by the investigator at the in-person visit. Subjects' sleep diaries recorded via the App. The App data and bracelet data were exported by technical support staff and stored at the Department of Respiratory and Critical Care Medicine in Peking University First Hospital. To ensure the completeness and accuracy of the survey data, all data were verified and accepted prior to entry.
- Entry and preservation: All original data can be entered only after de-identifying information, and will be summarized and preserved by the Department of Respiratory and Critical Care Medicine, Peking University First Hospital.
- Quality control and verification of data management: clinical data are entered into the database synchronously and independently in double copies. Random sampling of data is performed by data management staff. Lock the database after comparing with no errors.

3.9 Statistical Analysis

SPSS statistical software was used for data management and statistical analysis. Continuous variables were expressed as mean ± standard deviation or median (interquartile spacing), and count data were expressed as number of cases (percentage). Continuous variables were compared using t-test or rank sum test, and categorical variables were compared using chi-square test or Fisher's exact probability test. Mixed regression models will be used for longitudinal data analysis. Multifactor analysis will be used to compare differences in primary and secondary outcomes between the intervention and control groups. The intention-to-treat (ITT) analysis will be performed for the primary outcomes. p-values <0.05 are set as statistically significant.

IV. Participants Protection

1. This protocol is subject to approval by the Ethics Committee and informed consent signed by the participants.
2. Participants' interests.
   - The software intervention is delivered primarily through patient education and behavioral instruction, and the content is set according to the guideline-recommended first-line treatment protocol specifications. All subjects will receive sleep education to facilitate and benefit from self-management.
   - There will be up to 4 in-person visits during the study period, and subjects will be compensated for their transportation.
   - At the end of the study, all subjects who enroll and complete the study receive a study-issued smart bracelet.
3. Possible risks for the subjects: Theoretically, according to the characteristics of CBT-I treatment, there is a possibility of sleep deprivation or consequent induction of mood disorders during the sleep restriction treatment sessions. However, according to previous literature, no significant adverse effects have been reported in clinical studies with similar software abroad.

Moreover, the App will adjust the intensity of sleep restriction or discontinue the session if necessary based on daily patient feedback, reducing the risk of significant adverse effects.

4. Subjects may withdraw from the study at any time and for any reason, at their own discretion and without any consequences. The decision to withdraw a subject from the study may be made by the investigator for urgent medical reasons.
5. All raw data were de-identified and entered by the Department of Respiratory and Critical Care Medicine of Peking University First Hospital. The Shenzhen Zeen Health Technology Co., Ltd. was not involved in any statistical analysis or interpretation of the data.

Annexes

- Epworth Sleepiness Scale (ESS)
- Sleep Diary
- Insomnia severity index（ISI）
- Simplified Sleep Attitudes and Beliefs Questionnaire （DBAS-16）
- Fatigue severity scale (FSS)
- Quality of Life Questionnaire（SF-12）
- Generalized Anxiety Disorder Assessment （GAD-7）
- Patient Health Questionnaire(PHQ-9 Depression Screening)

---

[i] Ohayon MM. Epidemiology of insomnia: what we know and what we still need to learn. Sleep Medicine. 2002; 6(2):97–111.

[ii] Chinese Medical Association - Chinese Society of Neurology , Chinese Medical Association - Chinese Society of Neurology - Sleep Disorder Unit .Guidelines for the diagnosis and treatment of insomnia in Chinese adults (2017 edition). Chinese Journal of Neurology, 2018, 51(5): 324-335.

[iii] Morin CM, Bélanger L, LeBlanc M, Ivers H, Savard J, Espie CA, et al. The natural history of insomnia: a population-based 3-year longitudinal study. Arch Intern Med. 2009; 169(5):447–453. doi: 10.1001/ archinternmed.2008.610 PMID: 19273774

[iv] International Classification of Sleep Disorders, 3rd ed. Darien, Ill.: American Academy of Sleep Medi- cine; 2014.

[v] Association AP. Diagnostic and statistical manual of mental disorders, fifth edition (DSM-5). Arlington, VA: American Psychiatric Association, 2013.

[vi] Daley M, Morin CM, Leblanc M, et al. The economic burden of insomnia: direct and indirect costs for individuals with insomnia syndrome, insomnia symptoms, and good sleepers. Sleep, 2009, 32(1): 55-64.

[vii] Grandner MA, Jackson NJ, Izci-Balserak B, Gallagher RA, Murray-Bachmann R, Williams NJ, et al. Social and behavioral determinants of perceived insufficient sleep. Front Neurol. 2015; 6:112. doi: 10. 3389/fneur.2015.00112 PMID: 26097464

[viii] Markwald RR, Melanson EL, Smith MR, Higgins J, Perreault L, Eckel RH, et al. Impact of insufficient sleep on total daily energy expenditure, food intake, and weight gain. Proc Natl Acad Sci U S A. 2013; 110(14):5695–5700. doi: 10.1073/pnas.1216951110 PMID: 23479616

[ix] Everitt H, Baldwin DS, Stuart B, Lipinska G, Mayers A, Malizia AL, Manson CC, Wilson S. Antidepressants for insomnia in adults. Cochrane Database Syst Rev. 2018 May 14;5(5):CD010753. doi: 10.1002/14651858.CD010753.pub2. PMID: 29761479; PMCID: PMC6494576.

[x] Berry SD, Lee Y, Cai S, Dore DD. Nonbenzodiazepine sleep medication use and hip fractures in nursing home residents. JAMA Intern Med. 2013 May 13;173(9):754-61. doi: 10.1001/jamainternmed.2013.3795. PMID: 23460413; PMCID: PMC3676706.

[xi] Trauer JM, Qian MY, Doyle JS, Rajaratnam SM, Cunnington D. Cognitive Behavioral Therapy for Chronic Insomnia: A Systematic Review and Meta-analysis. Ann Intern Med. 2015 Aug 4;163(3):191-204. doi: 10.7326/M14-2841. PMID: 26054060.

[xii] van Straten A, van der Zweerde T, Kleiboer A, Cuijpers P, Morin CM, Lancee J. Cognitive and behavioral therapies in the treatment of insomnia: A meta-analysis. Sleep Med Rev. 2018 Apr;38:3-16. doi: 10.1016/j.smrv.2017.02.001. Epub 2017 Feb 9. PMID: 28392168.

[xiii] van der Zweerde T, Bisdounis L, Kyle SD, Lancee J, van Straten A. Cognitive behavioral therapy for insomnia: A meta-analysis of long-term effects in controlled studies. Sleep Med Rev. 2019 Dec;48:101208. doi: 10.1016/j.smrv.2019.08.002. Epub 2019 Aug 12. PMID: 31491656.

[xiv] Riemann D, Baglioni C, Bassetti C, Bjorvatn B, Dolenc Groselj L, Ellis JG, Espie CA, Garcia-Borreguero D, Gjerstad M, Gonçalves M, Hertenstein E, Jansson-Fröjmark M, Jennum PJ, Leger D, Nissen C, Parrino L, Paunio T, Pevernagie D, Verbraecken J, Weeß HG, Wichniak A, Zavalko I, Arnardottir ES, Deleanu OC, Strazisar B, Zoetmulder M, Spiegelhalder K. European guideline for the diagnosis and treatment of insomnia. J Sleep Res. 2017 Dec;26(6):675-700. doi: 10.1111/jsr.12594. Epub 2017 Sep 5. PMID: 28875581.

[xv] Qaseem A, Kansagara D, Forciea MA, Cooke M, Denberg TD; Clinical Guidelines Committee of the American College of Physicians. Management of Chronic Insomnia Disorder in Adults: A Clinical Practice Guideline From the American College of Physicians. Ann Intern Med. 2016 Jul 19;165(2):125-33. doi: 10.7326/M15-2175. Epub 2016 May 3. PMID: 27136449.

[xvi] Espie CA, MacMahon KM, Kelly HL, Broomfield NM, Douglas NJ, Engleman HM, McKinstry B, Morin CM, Walker A, Wilson P. Randomized clinical effectiveness trial of nurse-administered small-group cognitive behavior therapy for persistent insomnia in general practice. Sleep. 2007 May;30(5):574-84. doi: 10.1093/sleep/30.5.574. PMID: 17552372.

[xvii] Seyffert M, Lagisetty P, Landgraf J, Chopra V, Pfeiffer PN, Conte ML, et al. (2016) Internet-Delivered Cognitive Behavioral Therapy to Treat Insomnia: A Systematic Review and Meta-Analysis. PLoS ONE 11(2): e0149139. doi:10.1371/journal. pone.0149139

[xviii] Vedaa Ø, Kallestad H, Scott J, Smith O, Pallesen S, Morken G, Langsrud K, Gehrman P, Thorndike F, Ritterband L, Harvey AG, Stiles T, Sivertsen B. Effects of digital cognitive behavioural therapy for insomnia on insomnia severity: a large-scale randomised controlled trial. Lancet Digital Health 2020; 2: e397–406
