# Supplementary material for: Digital Cognitive Behavioral Therapy for Insomnia Using a Smartphone Application in China: A Pilot Randomized Clinical Trial
Source: JAMA Netw Open. 2023 Mar 27;6(3):e234866. doi: 10.1001/jamanetworkopen.2023.4866 (PMC10043748; doi:10.1001/jamanetworkopen.2023.4866)
Supplement: Supplement 2. — eTable 1. Contents of Each Phase of DCBT-I App eTable 2. Comparisons of DCBT-I vs Sleep Education ISI Scores in PP Analysis eTable 3. Comparison of ISI Response and Remission Between the DCBT-I and Sleep Education Groups eTable 4. Comparisons of Self-reported Scales Between the DCBT-I and Sleep Education Groups eTable 5. Comparisons of Smart Bracelet Measures Between the DCBT-I and Sleep Education Groups eFigure 1. Differences in ISI Scores Between the DCBT-I and Sleep Education Groups Over Time, Estimated From a Regression Model With a Random Slope eFigure 2. Screenshots of the DCBT-I App [file jamanetwopen-e234866-s002.pdf]

Supplemental Online Content

Zhang C, Liu Y, Guo X, Liu Y, Shen Y, Ma J. Digital cognitive behavioral therapy for insomnia using a smartphone application in China: a pilot randomized clinical trial. *JAMA Netw Open*. 2023;6(3):e234866. doi:10.1001/jamanetworkopen.2023.4866

eTable 1. Contents of Each Phase of DCBT-I App
eTable 2. Comparisons of DCBT-I vs Sleep Education ISI Scores in PP Analysis
eTable 3. Comparison of ISI Response and Remission Between the DCBT-I and Sleep Education Groups
eTable 4. Comparisons of Self-reported Scales Between the DCBT-I and Sleep Education Groups
eTable 5. Comparisons of Smart Bracelet Measures Between the DCBT-I and Sleep Education Groups
eFigure 1. Differences in ISI Scores Between the DCBT-I and Sleep Education Groups Over Time, Estimated From a Regression Model With a Random Slope
eFigure 2. Screenshots of the DCBT-I App

This supplemental material has been provided by the authors to give readers additional information about their work.

© 2023 Zhang C et al. *JAMA Network Open.*

eTable 1. Contents of Each Phase of DCBT-I App

Phases	Content
Phase 1: Familiarization with App operation and intervention processes	It begins with an introduction to resleep and CBT-I, followed by an explanation of how to use resleep and how the treatment works, as well as the treatment duration and its precautions for use. It also instructs participants to interact with the App and explains the sleep diary and the causes of insomnia. There is no intervention in this phase.
Phase 2: Sleep Restriction	It provides lectures on sleep restriction with its principles and effects. Then, the bedtime and wake-up time will be set. The time of sleep restriction will also be adjusted according to the changes in the participant's sleep-related indicators. During this period, the participant will be encouraged and reassured to improve treatment adherence. Sleep restriction starts on the 4th day of treatment and continues until the end of treatment.
Phase 3: Relaxation training	It first introduces relaxation training, explaining the purpose and role of relaxation training. Then, the audio instruction of positive meditation, breathing exercises, progressive muscle relaxation exercises are provided, and the time and methods of relaxation training are informed. Relaxation training starts on the 7th day of treatment and continues until the end of treatment
Phase 4: Stimulation control	It introduces the stimulation control, explaining the principles and effects. It provides the suggestion of activity options for participants when they are not in bed. It collects participants' feelings to give encouragement and reassurance and explains again the purpose of stimulation control.
Phase 5: Sleep hygiene	It introduces the content of sleep hygiene education, explains the principles to correct participants' poor sleep habits and establish helpful sleeping habits
Phase 6: Cognitive reconstruction	It explains some wrong perceptions about sleep, guides participants to establish correct perceptions about sleep and adjusts their mindset to reduce anxiety and worries about sleep
Phase 7: Consolidation and recovery	It summarizes the contents of the previous treatment phases to reinforce the impression and consolidate the treatment

© 2023 Zhang C et al. *JAMA Network Open.*

eTable 2. Comparisons of DCBT-I vs Sleep Education ISI Scores in PP analysis

	Difference test			
	**Sleep Education**	**dCBT-I**	**t**	***P***
Baseline	20.8 (3.3)	20.8(3.7)	0.059	0.953
Post Intervention	14.9 (4.9)	12.7(4.8)	1.946	0.056
1-month Follow-up	14.2 (5.0)	12.9(5.1)	1.099	0.276
3-month Follow-up	14.8 (5.6)	12.1(5.5)	2.041	0.045*
6-month Follow-up	14.1 (5.7)	11.5(5.4)	1.961	0.051

Data were shown as mean (standard deviation)

**P*<0.05

eTable 3. Comparison of ISI Response and Remission Between the DCBT-I and Sleep Education Groups

**Test Variables**	**Sleep Education**	**dCBT-I**	$\chi 2$	***P***
**ISI Responders (Reductions≥8) (Number of Persons [%])**				
Post Intervention	12(30.8)	25(65.8)	9.456	0.002*
1-mo Follow-up	14(35.9)	21(55.3)	2.911	0.088
3-mo Follow-up	14(35.9)	24(63.2)	5.722	0.017*
6-mo Follow-up	17(43.6)	22(57.9)	1.576	0.209
**ISI Remitters (ISI<8) (Number of Persons [%])**				
Post Intervention	3(7.7)	7(18.4)	1.960	0.161
1-mo Follow-up	4(10.3)	7(18.4)	1.048	0.306
3-mo Follow-up	5(12.8)	8(21.1)	0.929	0.335
6-mo Follow-up	4(10.3)	9(23.7)	2.473	0.116

ISI reduction ≥8 points were defined as treatment responders (ISI responders). ISI<8 was used defined for ISI remission (ISI remitter). **P*<0.05

© 2023 Zhang C et al. *JAMA Network Open.*

eTable 4. Comparisons of Self-reported Scales Between the DCBT-I and Sleep Education Groups

Scales	Difference test				
	**Sleep Education**	**dCBT-I**	**t values**	**P**	**Cohen's d (95% CI)**
**DBAS-16**					
Baseline	109.6(21.0)	107.1(23.6)	0.493	0.623	--
Post Intervention	99.4(32.7)	96.3(30.0)	0.428	0.670	0.098 (-0.350 to 0.544)
1-month Follow-up	99.0(30.9)	91.0(30.7)	1.137	0.259	0.261(-0.192 to 0.712)
3-month Follow-up	96.9(33.3)	93.5(32.0)	0.455	0.650	0.105(-0.348 to 0.558)
6-month Follow-up	93.8(31.2)	85.6(29.1)	1.140	0.258	0.269(-0.196 to 0.732)
**FSS**					
Baseline	45.2(16.8)	43.7(11.7)	0.436	0.664	--
Post Intervention	41.2(20.3)	38.0(15.2)	0.789	0.433	0.180(-0.269 to 0.627)
1-month Follow-up	41.9(18.0)	38.1(15.8)	0.967	0.337	0.222(-0.230 to 0.672)
3-month Follow-up	40.1(18.0)	38.4(16.4)	0.422	0.675	0.097(-0.356 to 0.550)
6-month Follow-up	37.7(18.0)	35.5(17.5)	0.537	0.593	0.127(-0.336 to 0.589)
**GAD-7**					
Baseline	5.9(4.5)	5.9(3.7)	0.057	0.955	--
Post Intervention	4.4(3.3)	4.1(3.2)	0.447	0.656	0.102(-0.345 to 0.549)
1-month Follow-up	4.9(3.8)	3.9(3.7)	1.069	0.289	0.245(-0.207 to 0.696)
3-month Follow-up	5.1(3.9)	3.4(3.3)	2.051	0.044*	0.474(0.013 to 0.931)
6-month Follow-up	4.6(3.8)	2.9(3.0)	2.225	0.029*	0.524(0.053 to 0.993)
**PHQ-9**					
Baseline	10.9(5.6)	10.2(4.1)	0.704	0.483	--
Post Intervention	7.9(4.8)	5.9(3.8)	1.989	0.050	0.453(-0.000 to 0.904)
1-month Follow-up	7.4(4.9)	6.5(4.2)	0.878	0.383	0.202(-0.250 to 0.652)
3-month Follow-up	8.2(5.0)	5.6(3.8)	2.527	0.014*	0.584(0.119 to 1.044)
6-month Follow-up	7.6(5.9)	5.5(3.3)	1.857	0.067	0.438(-0.031 to 0.904)
**SF-12 MCS**					
Baseline	39.5(9.5)	40.5(8.6)	-0.497	0.621	--
Post Intervention	40.7(9.0)	42.7(9.1)	-0.958	0.341	-0.218(-0.666 to 0.230)
1-month Follow-up	40.5(8.8)	43.0(9.4)	-1.195	0.236	-0.274(-0.725 to 0.179)
3-month Follow-up	40.5(9.0)	44.9(7.3)	-2.289	0.025*	-0.529(-0.988 to -0.066)
6-month Follow-up	42.8(9.7)	45.4(8.6)	-1.222	0.226	-0.286(-0.746 to 0.176)
**SF-12 PCS**					
Baseline	20.6(5.3)	21.7(6.2)	-0.852	0.397	--
Post Intervention	20.7(6.0)	21.4(4.9)	-0.530	0.598	-0.121(-0.568 to 0.327)
1-month Follow-up	21.1(6.0)	21.5(5.6)	-0.276	0.783	-0.063(-0.513 to 0.387)
3-month Follow-up	21.2(6.0)	21.0(6.5)	0.143	0.887	0.033(-0.420 to 0.486)
6-month Follow-up	20.9(5.5)	21.3(7.9)	-0.254	0.800	-0.059(-0.518 to 0.400)

PCS, Physical Component Scores; MCS, Mental Component Scores.

Data were shown as mean (standard deviation). *p<0·05

© 2023 Zhang C et al. *JAMA Network Open.*

eTable 5. Comparisons of Smart Bracelet Measures Between the DCBT-I and Sleep Education Groups

Smart bracelet measures	Difference test				
	Sleep Education	dCBT-I	t value	P	Cohen d (95% CI)
**Total sleep time (min)**					
Baseline	429.0(68.5)	449.0(75.7)	-1.182	0.241	--
Post Intervention	407.1(122.0)	434.3(100.1)	-1.019	0.312	-0.244(-0.713 to 0.228)
1-month Follow-up	414.8(85.3)	444.4(86.9)	-1.468	0.147	-0.344(-0.805 to 0.120)
3-month Follow-up	395.8(91.8)	448.4(71.3)	-2.591	0.012*	-0.643(-1.14 to -0.142)
6-month Follow-up	453.0(71.4)	476.1(115.0)	-0.939	0.352	-0.240(-0.743 to 0.264)
**Time of Waking up During Sleep (min)**					
Baseline	12.2(17.4)	10.3(14)	0.521	0.604	--
Post Intervention	8.6(9.8)	10.4(11.7)	-0.701	0.486	-0.168(-0.636 to 0.302)
1-month Follow-up	10.1(9.0)	9.3(11.2)	0.311	0.757	0.073(-0.386 to 0.532)
3-month Follow-up	11.4(11.9)	6.8(8.0)	1.815	0.074	0.451(-0.044 to 0.942)
6-month Follow-up	13.0(14.6)	9.3(11.8)	1.096	0.277	0.281(-0.225 to 0.784)

Data were shown as mean (standard deviation). *p<0·05

© 2023 Zhang C et al. *JAMA Network Open.*

eFigure 1. Differences in ISI Scores Between the DCBT-I and Sleep Education Groups Over Time, Estimated From a Regression Model With a Random Slope

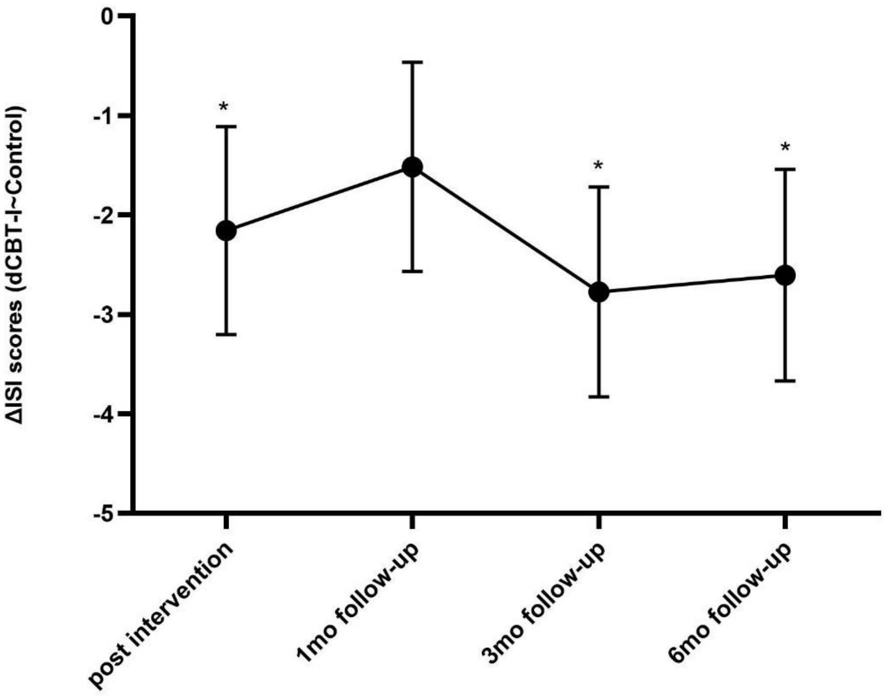

*P<0.05 for group×time interaction effect. The error bars were shown for the standard errors of the estimations.

'ΔISI': The differences in ISI scores; 'mo': month.

ΔISI was -2.16 (95%CI: -4.21, -0.11), -1.52 (95%CI: -3.58, 0.55), -2.77(95%CI: -4.84, -0.70), -2.61 (95%CI: -4.69, -0.52) for post-intervention, 1 mo follow-up, 3 mo follow-up and 6 mo follow-up respectively.

(The group x time interactions were quantified by a regression model with a random slope, which is parameterized as

Scorei, t ~ giβ1 + tβ2 + (t × gi)β3 + (i)'

where i and t denote the indexes for subject and visit (baseline, post-treatment and follow-ups), respectively; gi denotes the two groups (sleep education or dCBT-1 treatment); β3 denotes the effect of the trial; (i)' denotes the subject-specific slopes to capture the auto-correlations between the repeated measurements. We estimated β3 and the corresponding 95% confidence intervals.

© 2023 Zhang C et al. *JAMA Network Open.*

eFigure 2. Screenshots of the DCBT-I App

dCBT-I group：

1. Resleep is a virtual sleep therapist that simulates an offline patient-therapist communication scenario.

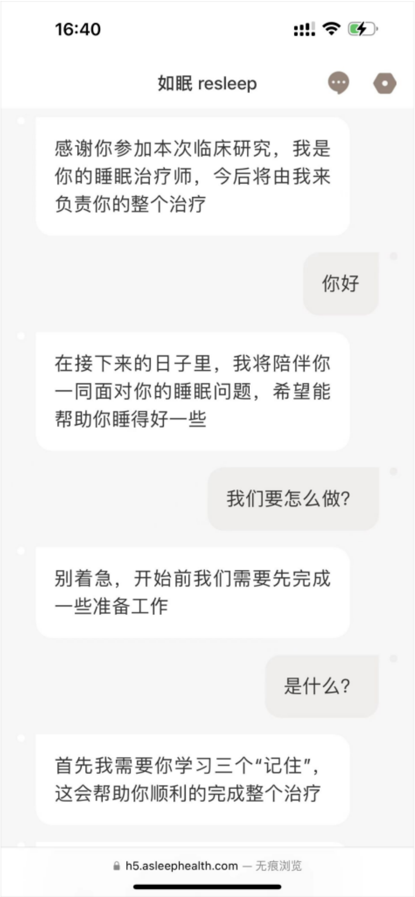

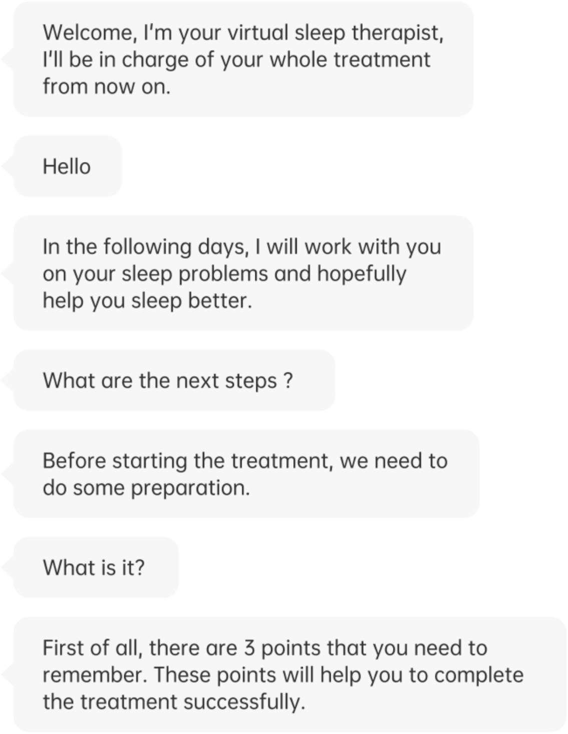

(Translation)

© 2023 Zhang C et al. *JAMA Network Open.*

2. Writing a sleep diary through a dialogue format.

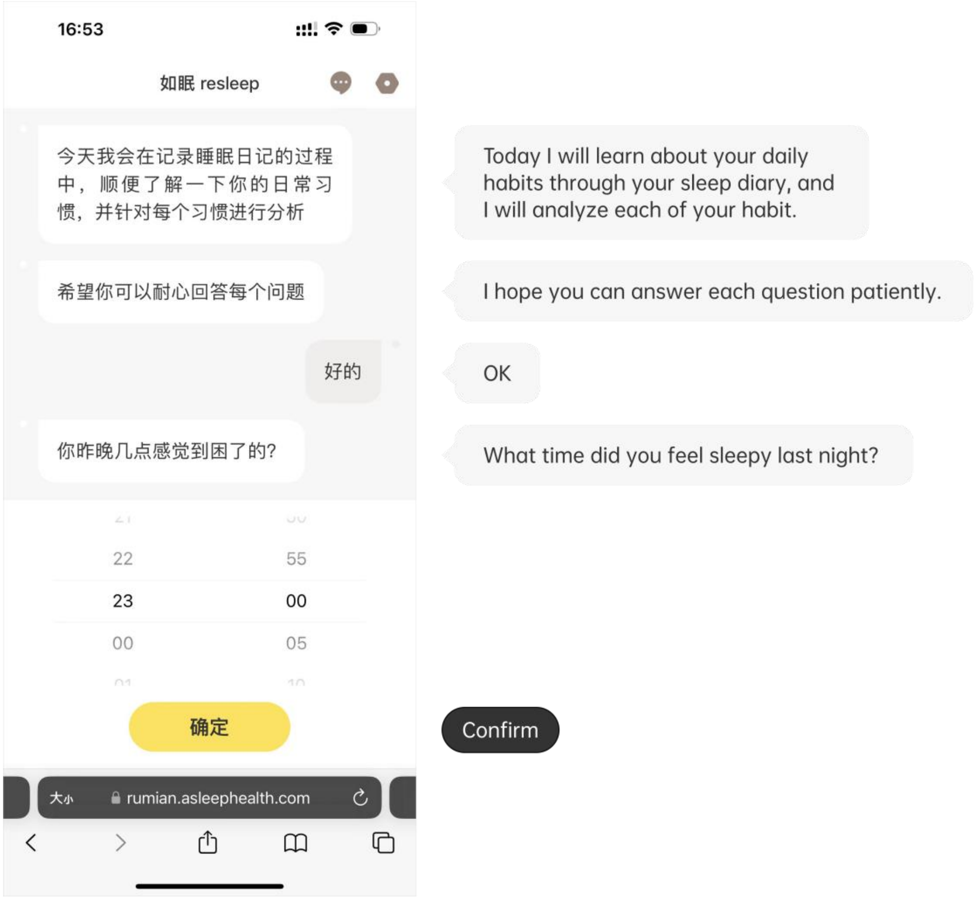

© 2023 Zhang C et al. *JAMA Network Open.*

3. During the conversation with the participants, resleep provides a variety of options for the participant to choose the one that best fits his or her situation. For the question about “what time do you get up？”, we rephrase the question "what time do you leave the bed" to make it easier to understand for the participants.

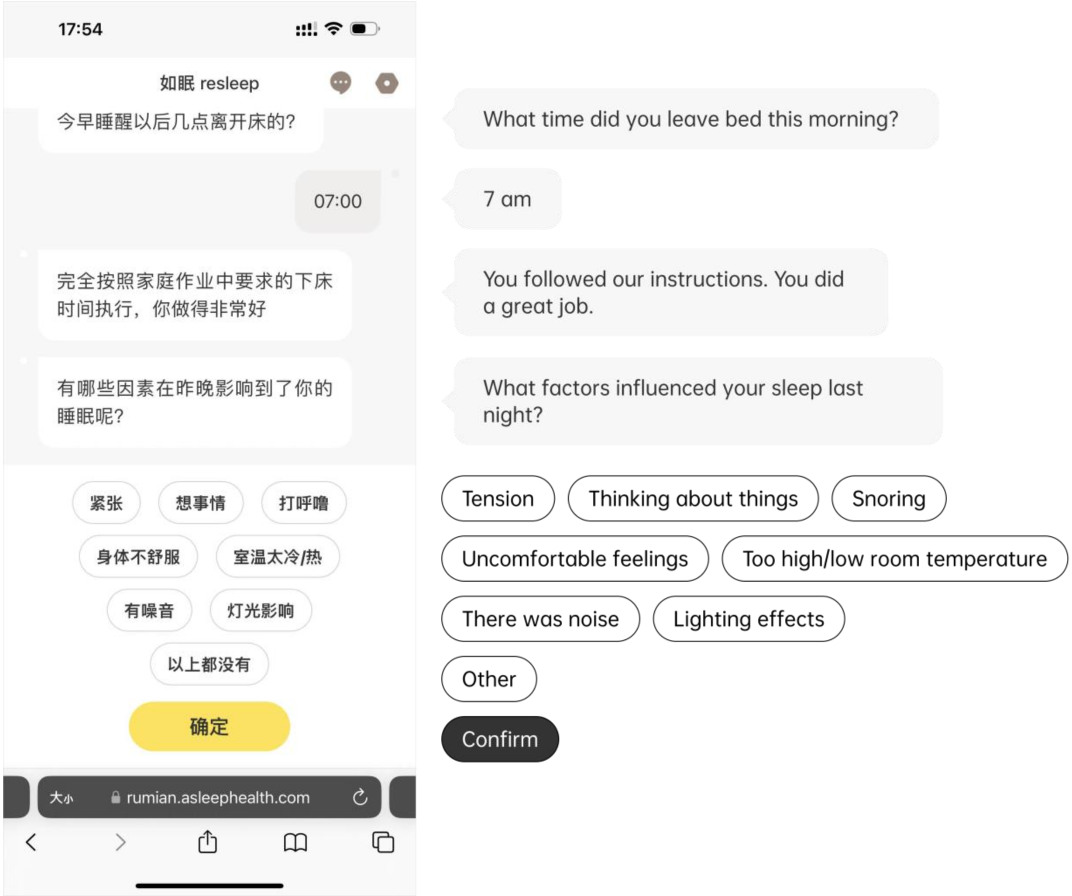

© 2023 Zhang C et al. *JAMA Network Open.*

4. Resleep provides the participant sleep hygiene education and explains its principles and roles.

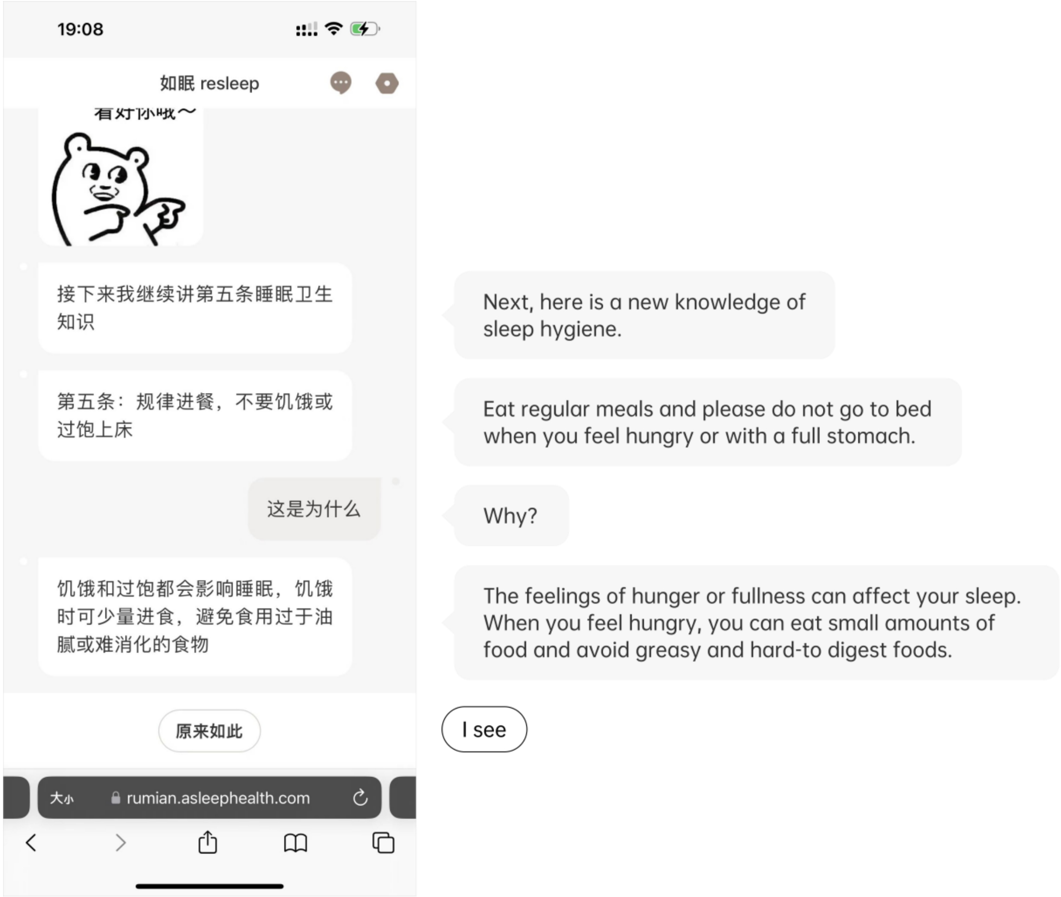

© 2023 Zhang C et al. *JAMA Network Open.*

5. Using the metaphor of "thinking of plums to quench thirst" (a Chinese idiom) to explain stimulus control.

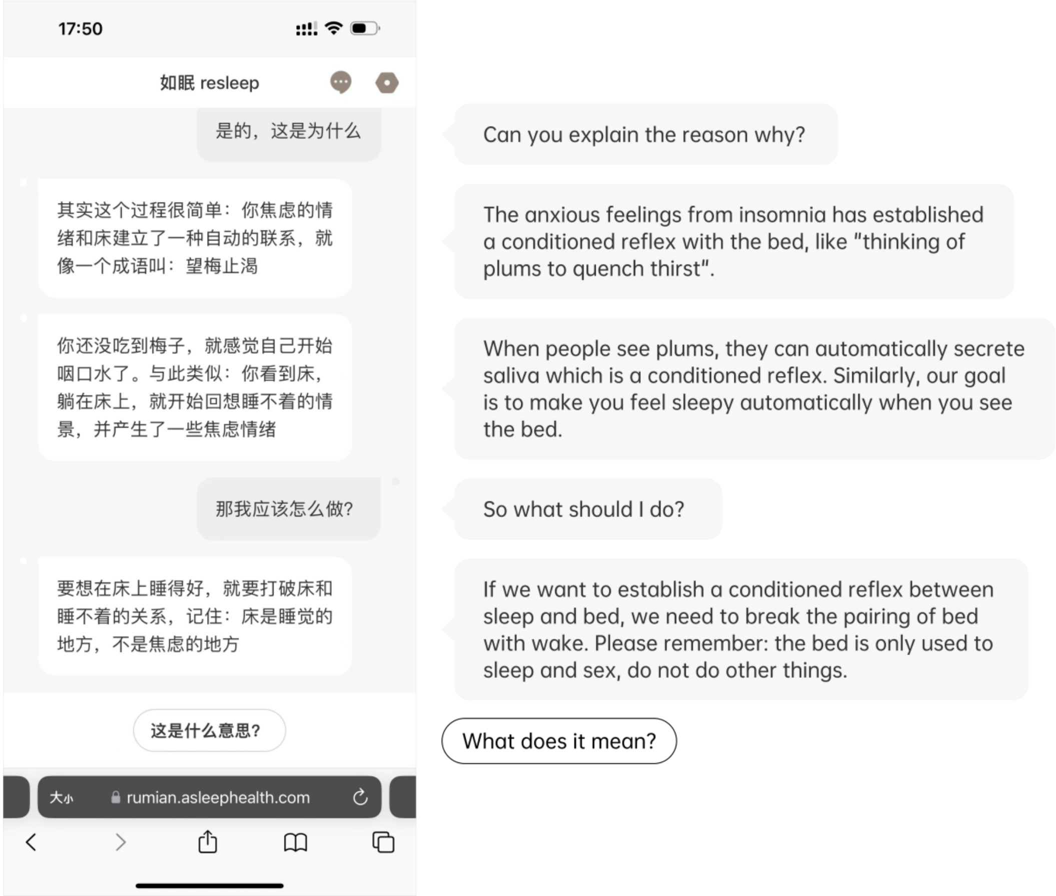

© 2023 Zhang C et al. *JAMA Network Open.*

6. In the stimulus control section, if participants are unable to fall asleep and leave the bed at night, we suggest several Chinese-culturally activities to help participants feeling calm and stay focused.

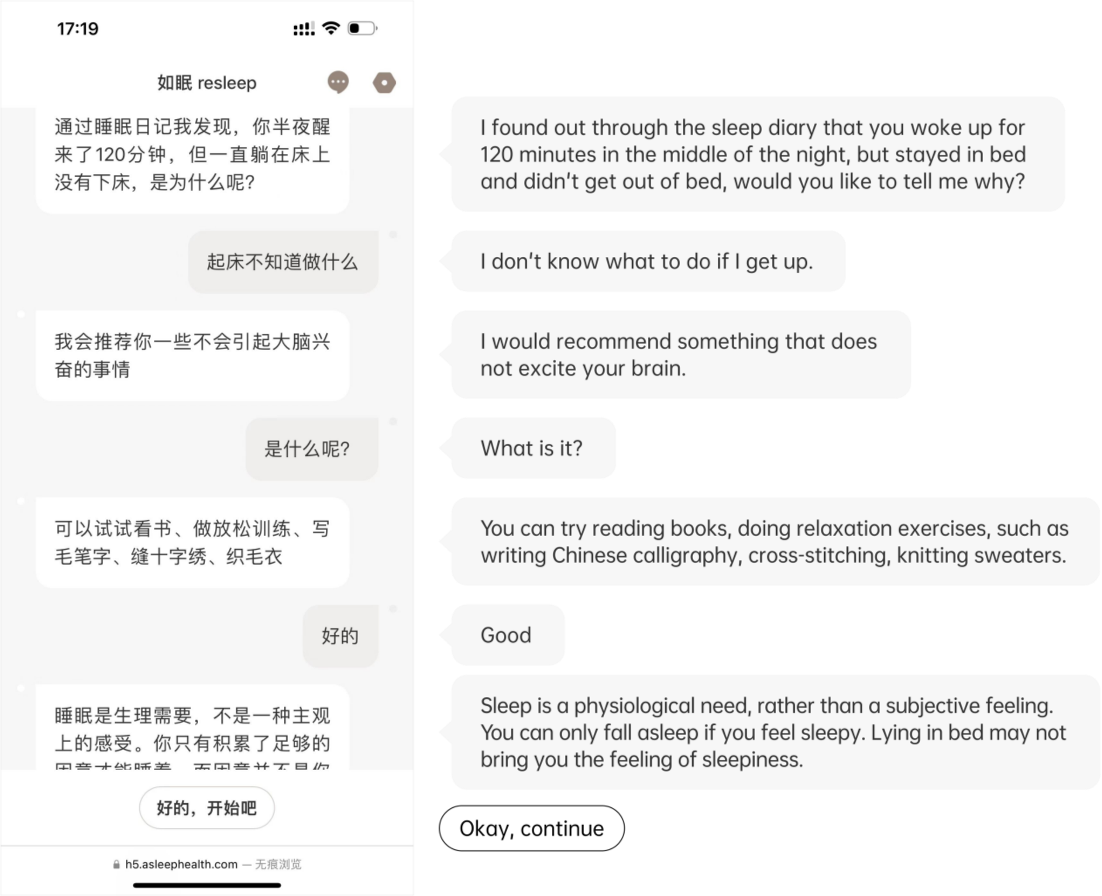

© 2023 Zhang C et al. *JAMA Network Open.*

7. Many Chinese people believe that they have to go to bed at 23:00 and sleep beyond this time is unhealthy. Therefore, they will lie in bed to rest even if they are not sleepy. Resleep intervenes this misperception and behavior.

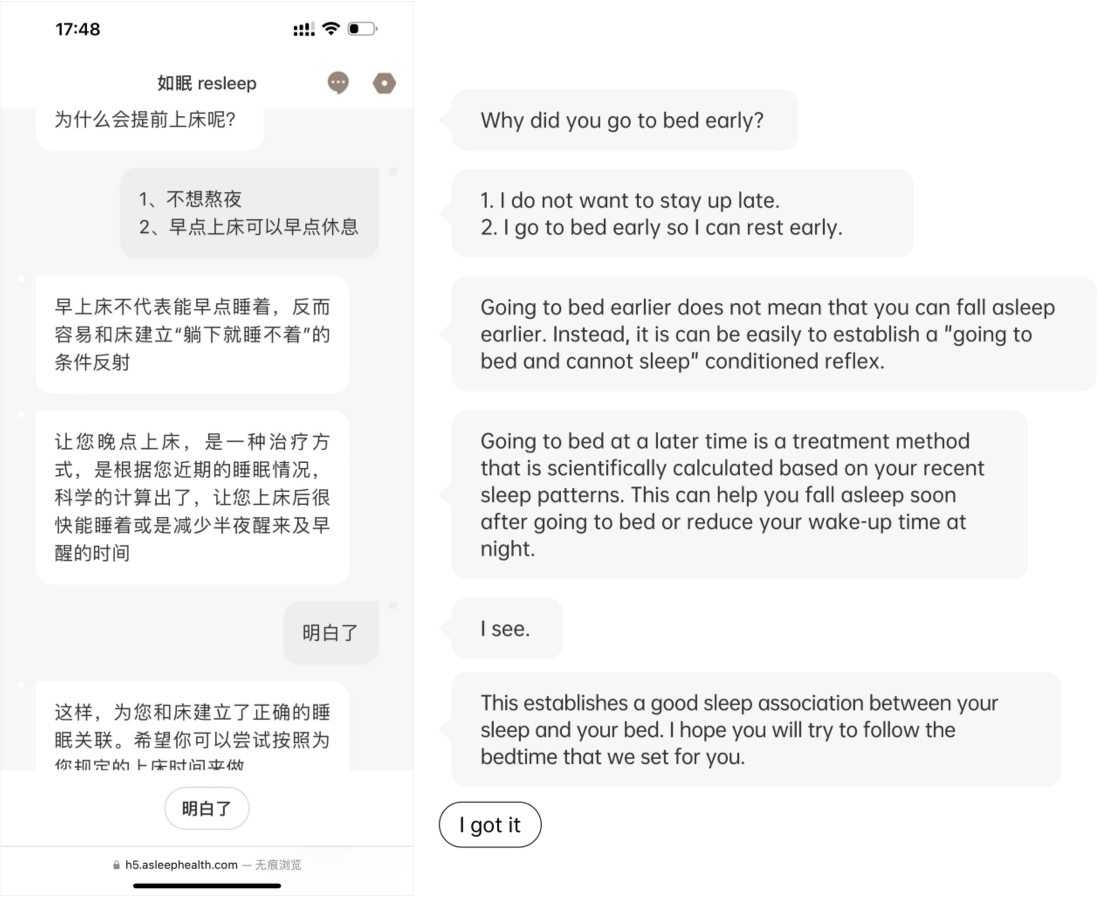

8. As participants follow the instructions, resleep will provide positive affirmation and encouragement comments. A daily summary of your sleep yesterday will be presented to help the participant gain better understanding of their sleep changes.

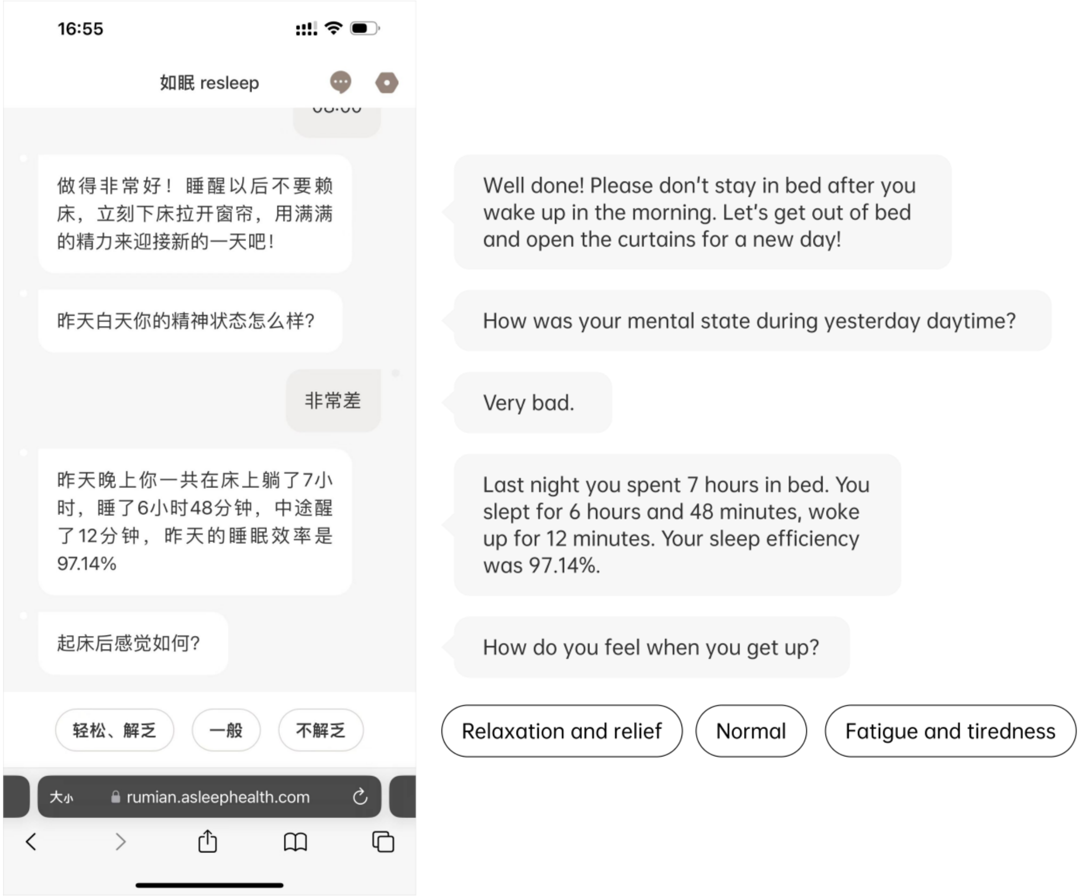

© 2023 Zhang C et al. *JAMA Network Open.*

9. At the end of conversation every day, resleep provides CBT-I treatment by assigning a homework. For many Chinese people who have napping habits, resleep recommends the participants to do relaxation exercises at noon instead of taking a nap.

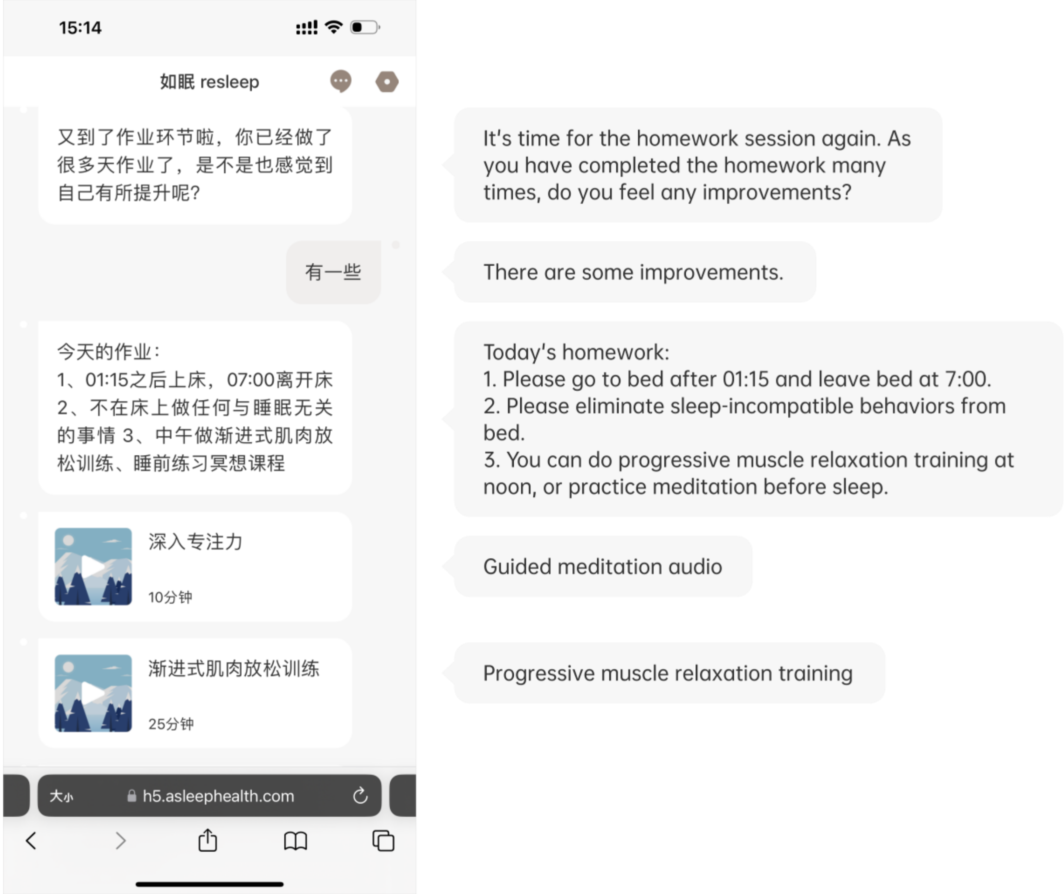

© 2023 Zhang C et al. *JAMA Network Open.*

10. Resleep adjusts the bedtime for each participant based on their sleep pattern changes.

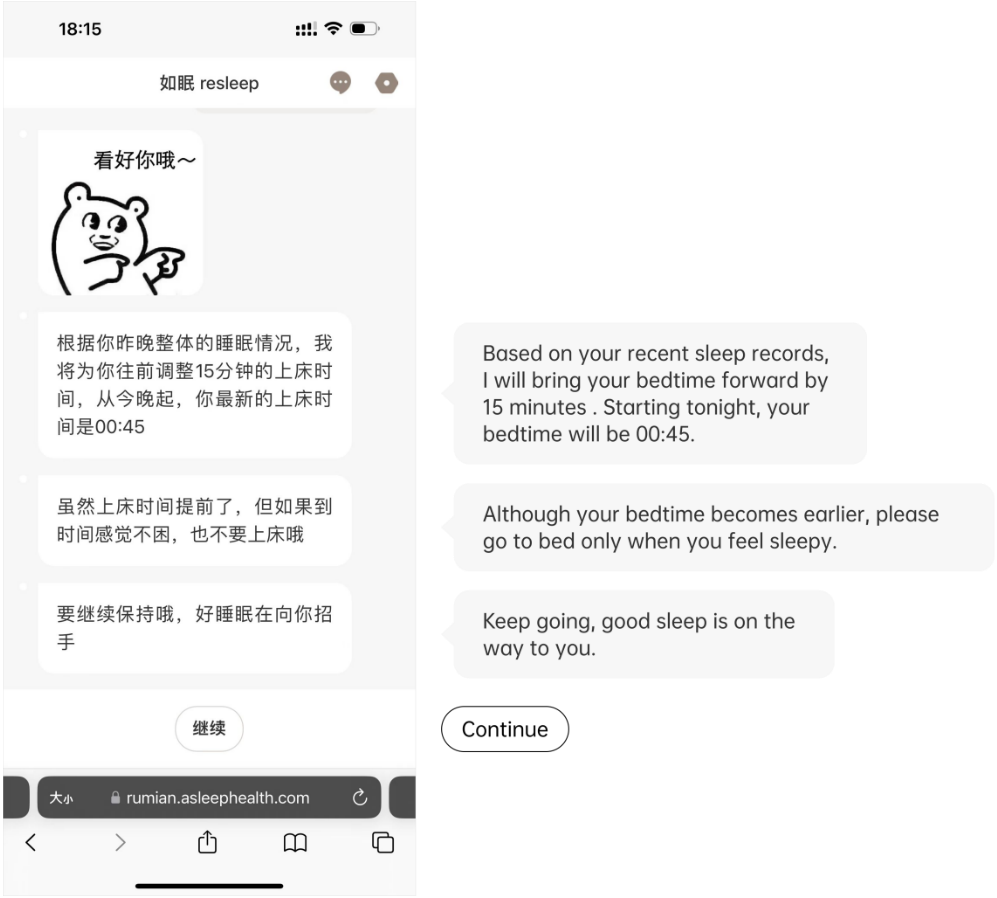

© 2023 Zhang C et al. *JAMA Network Open.*

Control group：

1. Resleep is a virtual sleep therapist that simulates an offline patient-therapist communication scenario.

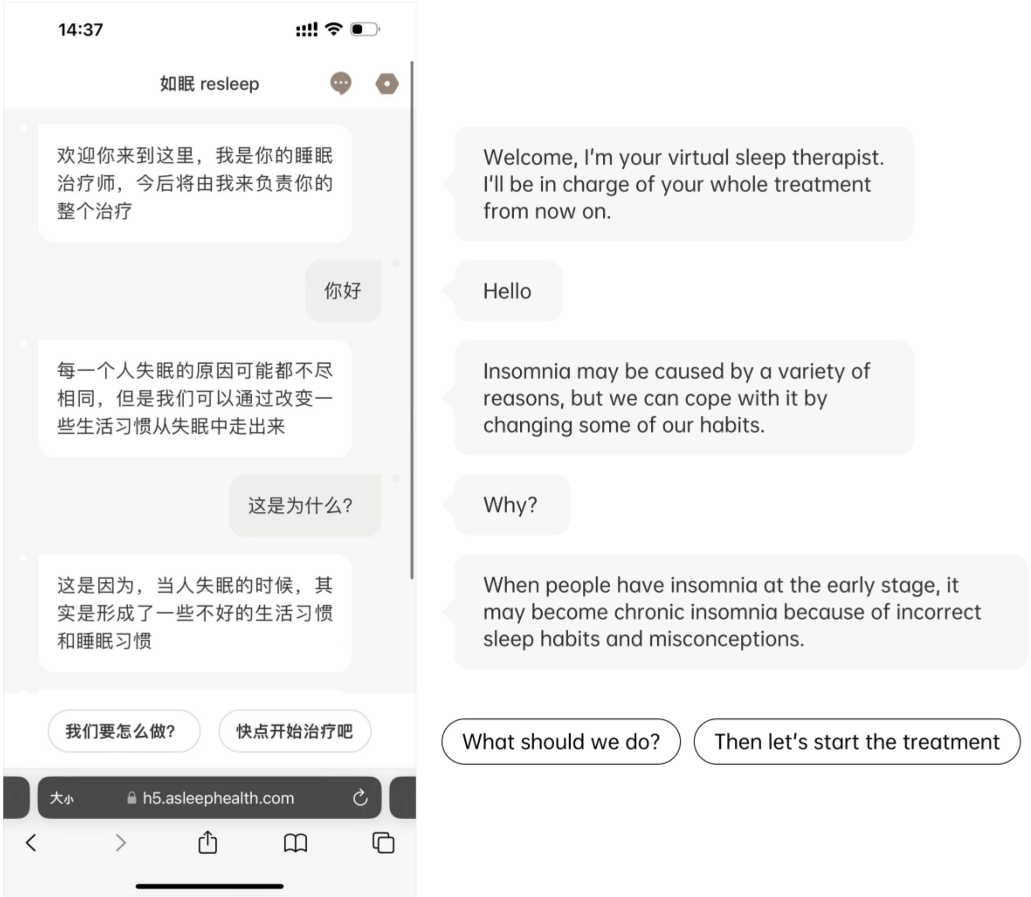

© 2023 Zhang C et al. *JAMA Network Open.*

2. Sleep diary is recorded daily through a dialogue format with the same interface as the treatment group.

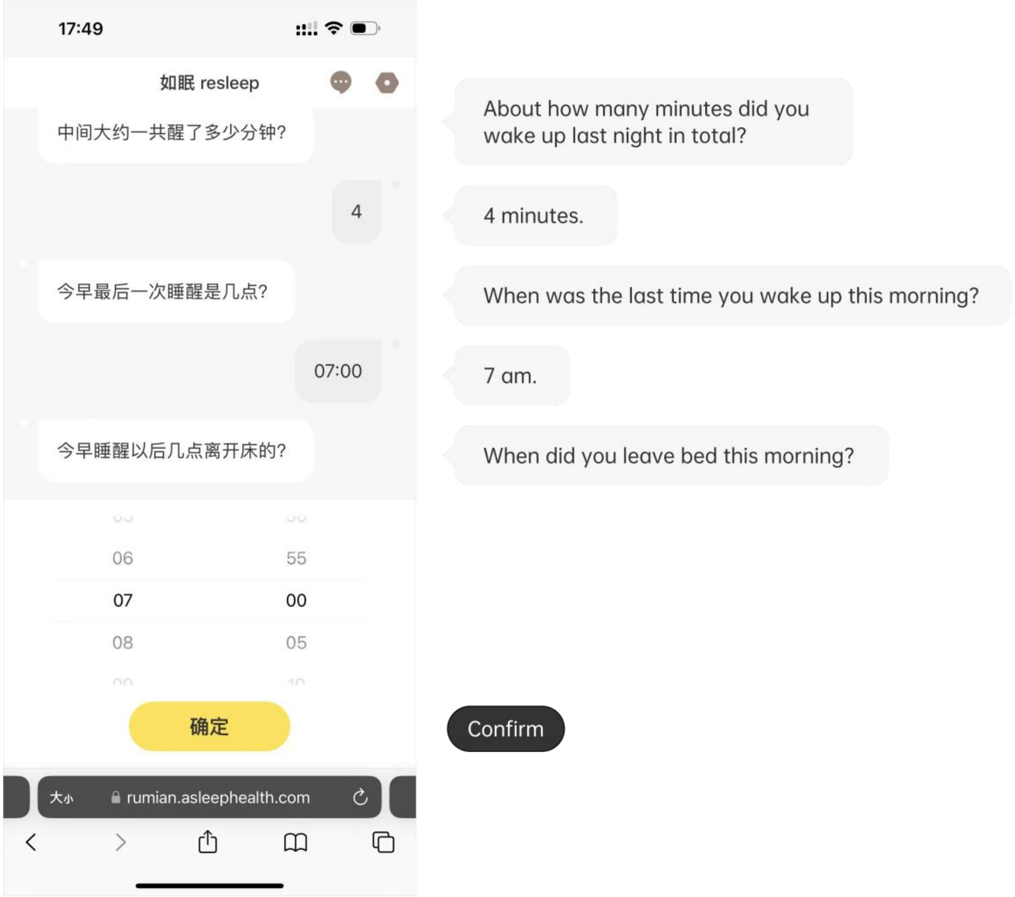

© 2023 Zhang C et al. *JAMA Network Open.*

3. Sleep hygiene education is provided daily to control group subjects.

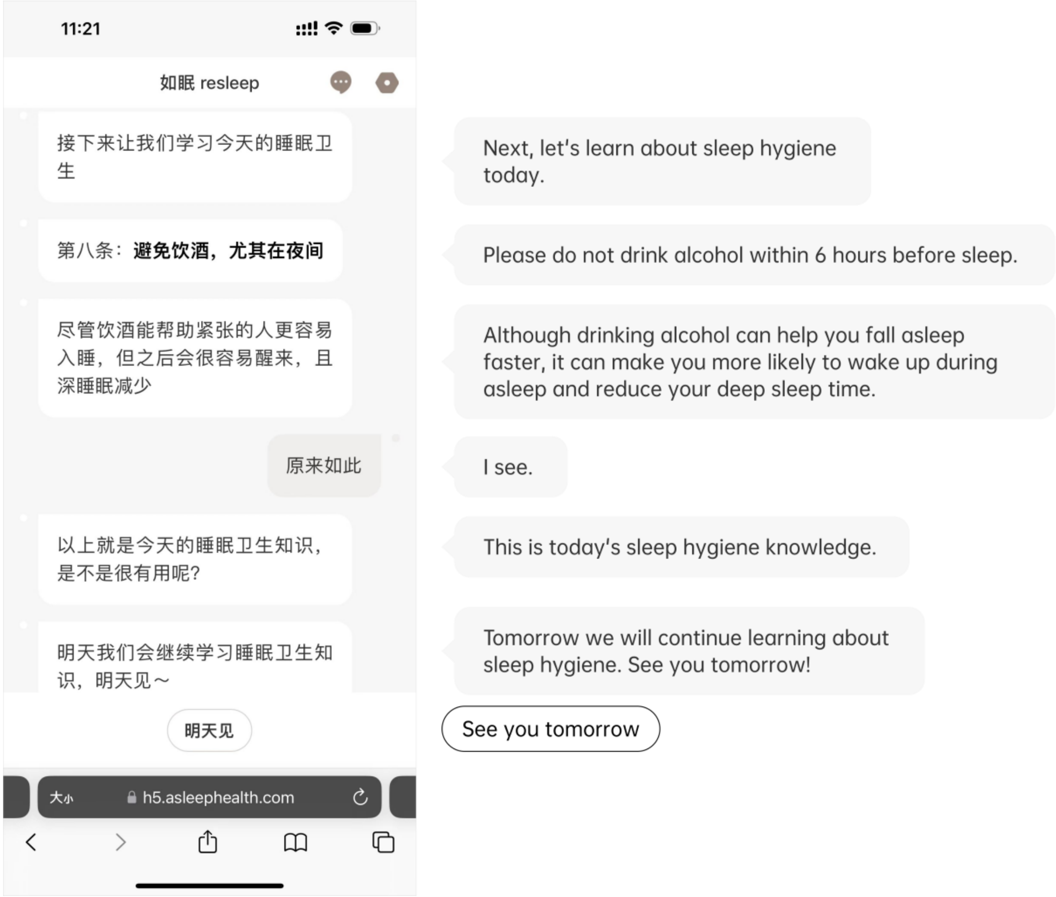

© 2023 Zhang C et al. *JAMA Network Open.*
