# Supplementary material for: Digital Cognitive Behavioral Therapy for Insomnia Using a Smartphone Application in China: A Pilot Randomized Clinical Trial
Source: JAMA Netw Open. 2023 Mar 27;6(3):e234866. doi: 10.1001/jamanetworkopen.2023.4866 (PMC10043748; doi:10.1001/jamanetworkopen.2023.4866)
Supplement: Supplement 3. — Data Sharing Statement [file jamanetwopen-e234866-s003.pdf]

Data Sharing Statement

Zhang. Digital Cognitive Behavioral Therapy for Insomnia Using a Smartphone Application in China. *JAMA Netw Open*. Published March 27, 2023.
doi:10.1001/jamanetworkopen.2023.4866

Data
Data available: Yes
Data types: Deidentified participant data
How to access data: The data that support the findings of this study are available from the corresponding author upon reasonable request. majjmail@163.com
When available: With publication

Supporting Documents
Document types: None

Additional Information
Who can access the data: Data for primary and secondary outcomes used for analysis
Types of analyses: upon reasonable reques
Mechanisms of data availability: with investigator support
